# Trends and demographic differences in interpersonal violence against children in sub-Saharan Africa: findings from the 1990–2019 Global Burden of Disease Study

**DOI:** 10.1136/bmjopen-2023-083070

**Published:** 2025-04-28

**Authors:** Sergio Nhassengo, Lucie Laflamme, Mathilde Sengoelge, GBD 2019 SSA Child Interpersonal Violence, Sergio Keita Nhassengo

**Affiliations:** 1 Department of Global Public Health, Karolinska Institutet, Stockholm, Sweden; 2 Eduardo Mondlane University, Maputo, Mozambique; 3 Institute for Social and Health Sciences, Pretoria, South Africa; 4 University for Continuing Education Krems, Krems, Niederösterreich, Austria

**Keywords:** Health Equity, Mortality, Child protection, Gender-Based Violence

## Abstract

**Abstract:**

**Objectives:**

To analyse the past 30-year trends in mortality and morbidity of interpersonal violence against children, its demographic distribution and correlation with specific risk factors.

**Design:**

Ecological study at the country and regional level.

**Setting:**

46 countries and 4 subregions of sub-Saharan Africa (SSA): Central, Eastern, Southern and Western.

**Participants:**

Children aged 0–19 years old.

**Primary and secondary outcome measures:**

Trends in mortality rates and disability-adjusted life-years (DALYs) attributed to interpersonal violence injuries in children; correlation between socio-demographic index (SDI)/alcohol consumption per capita and child interpersonal violence.

**Results:**

Deaths and DALYs per 100 000 population from child violence-related injuries in SSA declined from 4.0 (95% uncertainty interval (UI): 3.3–4.9) to 3.1 (95% UI: 2.3 to 3.9) and 334.9 (95% UI: 276.4 to 407.7) to 260.3 (95% UI: 197.9 to 321.9) respectively from 1990 to 2019 (reductions of 22.5% and 22.3%). Southern SSA had the highest deaths/DALYs rates for each type of physical violence (sharp object/firearm/other) and Central SSA for sexual violence. Alcohol consumption correlated significantly with deaths and DALYs, but SDI showed a non-significant correlation.

**Conclusions:**

Rates of child interpersonal violence deaths and DALYs decreased from 2009 to 2019 in SSA, driven by remarkable decreases in the Southern subregion. Understanding the determinants of these downward trends and implementation of policies targeting known risk factors like alcohol consumption may pave the way for enhanced child safety protection. Further curbing the disparities between countries and subregions necessitates long-term commitment to evidence-based action plans.

**STRENGTHS AND LIMITATIONS OF THIS STUDY**

- A primary strength of this study is the use of the most reliable burden of disease estimates with estimates of uncertainty that allow for trend compilations and regional comparisons for different forms of violence against children.
- Several forms of violence and outcome measures are considered that provide a better insight into the magnitude of violence against children and how differentially it affects boys and girls of various ages.
- The study results suffer from an underestimation bias, one that is inherent in any register-based estimation of the level, trends and consequences of violence.
- The study lacks data about certain forms of violence reported in the region, including female genital mutilation and psychological violence.
- Several documented drivers of violence against children have not been studied, including gender inequality, gun control policy, harmful traditional practices and law enforcement as regards to child protection.

## Introduction

 Violence against children is a global public health issue,^1^ and its prevention is advocated for in the Convention on the Rights of the Child—a right to freedom from violence—and in several targets of the Sustainable Development Goals, including, for example, Target 5.2 “eliminate all forms of violence against women and girls”, Target 16.1 “significantly reduce all forms of violence and related death rates everywhere” and Target 16.2 “end abuse, exploitation, trafficking and all forms of violence against children”. Yet, an estimated one out of two children, or 1 billion children, suffer some form of violence annually^1^; this includes 1.3 billion who sustain physical violence; 100 000 children who are victims of homicide; and 120 million girls who have suffered some form of forced sexual contact before the age of 20.^2^ Exposure to violence early in life is associated with multiple negative physical and mental health outcomes, such as a range of mental disorders, drug use, suicide attempts, sexually transmitted infections and risky sexual behaviour,^3^ often occurring simultaneously.^4^ The nature of the consequences sustained varies depending on the type of violence experience, the child’s age and gender, and both within and between countries, all to the detriment of the less well-off.^5^

This study adopted a socio-ecological framework to examine the multifaceted epidemiology aspects and risk factors of child interpersonal violence at the ecological level. While the framework includes a range of complex interplaying factors at different levels,^6^ this study focused on regional-level/country-level rates and drivers of child interpersonal violence to contribute to the development of country-level policies and action plans for the prevention of violence against children.^7^

Country-level determinants of violence against children include socio-economic circumstances and inequalities, cultural values and norms,^8^ as well as acknowledged injury risk factors.^9^ Low-income and middle-income countries tend to have higher rates of child interpersonal violence and face constraints in allocation resources in child protection and childcare services.^10^ At the micro-level, socio-economic disadvantages such as insufficient food, housing or healthcare disrupt parents’ caregiving abilities due to family deprivation; this may increase children’s risk of exposure to interpersonal violence.^11^ Also, high-income countries tend to have more focused interventions for prevention, response and treatment.^12^ In addition, the recent WHO report on alcohol and health points to alcohol as a significant contributory factor to child maltreatment.^13^ Alcohol use in the caregivers increases a child’s risk of experiencing violence as it may reduce parents’ ability to provide a supportive, nurturing and protective environment.^14^,^16^ To date, studies on violence against children published in the scientific literature are systematic reviews at the global level^17 18^ or region-specific.^19^ Many are focused on only one type of violence, for example, child sexual abuse, and based on combining different data sources. Overall, these studies show a similar trend of decreasing rates of prevalence of child violence in most recent years.^2 20^ Studies in Africa and the sub-Saharan African (SSA) region are few, despite the fact that they represent approximately 42% of the population and by 2026 onwards SSA will be the region with the greatest number of children under 18.^21^ SSA specifically has one of the world’s highest rates of violence against children, with estimates suggesting that 50% of children 2–14 years old and 51% of children 15–17 years old experienced violence annually.^17^ Yet, a systematic analysis of long-term country and SSA regional level trends is lacking, as well as an investigation that encompasses children of all ages by various types of violence and both fatal and non-fatal outcomes. The current study aims to shed light on mortality and morbidity trends of violence to children in SSA compared with global rates. We also investigated the correlation between country income level, country socio-demographic index, alcohol consumption and violence.

## Methods

### Global Burden of Disease 2019 Study and additional data source

This is an ecological study based on a systematic analysis of Global Burden of Disease (GBD) data from 1990 to 2019 (latest data available at time of study), whose analytic framework, methods and data sources have been provided in a previous publication.^22^ The study analyses mortality and morbidity data from the 46 countries and 4 subregions of SSA (Central, Eastern, Southern and Western) as defined by the GBD Study 2019 (see the countries per subregion in online supplemental table S1).

The World Bank economic-level classification was used to assess the correlation between child interpersonal violence and SSA countries income level based on gross national income per capita (classifications in online supplemental table S2).^23^ In the GBD Study 2019, SSA had 22 low-income, 19 lower-middle-income and 5 upper-middle-income countries (see breakdown in online supplemental tables S1 and S2).

### Definitions

#### Interpersonal violence

The GBD defines interpersonal violence as death or disability from intentional use of physical force or power, threatened or actual, from another person or group not including military or police forces (International Classification of Diseases, ninth revision (ICD-9): E960–E969; ICD-10: X85–Y08).^24^ In the GBD Study, interpersonal violence is a level 3 cause and encompasses four level 4 causes: (1) physical violence by firearm; (2) physical violence by a sharp object; (3) physical violence by other means and (4) sexual violence^24^ (classifications and definitions are provided in online supplemental table S3).

#### Disability-adjusted life-years

Disability-adjusted life-years (DALYs) are a commonly used metric that combines the sum of years lost due to premature death and years lived with disability to estimate the total number of years lost due to specific causes and risk factors. One DALY equals one lost year of healthy life.^24^

#### Socio-demographic index

Socio-demographic index (SDI) is an index created by the GBD Study that measures and identifies where countries or other geographical areas sit on the spectrum of development. SDI is based on a principal components analysis of educational attainment, average age of the population, income per capita and total fertility.^22^ The SDI has a value that ranges from 0 to 1, with 0 representing the lowest educational attainment, lowest income per capita and highest fertility under age 25 years; 1 represents the highest educational attainment, highest income per capita and lowest fertility under 25 years. Countries and territories are grouped into five quintiles of high, high-middle, middle, low-middle; and low SDI based on their values.^25^ In 2019, SSA had 26 countries with low SDI, 15 low-middle SDI and 5 middle SDI (see breakdown in online supplemental tables S1, S2).

### Patient and public involvement

Patients and/or the public were not involved in this study based on an ecological design of data at country level. The study was informed by the priorities of hospital staff at the Central Hospital of Maputo, Mozambique, who treat child victims of interpersonal violence and are striving to address this public health problem in Mozambique and the region. The research findings will be disseminated to the public via international conferences targeting researchers in injury prevention as well as regional and national conferences in SSA through the authors. This study complies with the Guidelines for Accurate and Transparent Health Estimates Reporting recommendations (online supplemental table S4).

### Data analysis

GBD 2009–2019 estimates analysed for this study are available on the Global Health Data Exchange (GHDx, http://ghdx.healthdata.org/gbd-results-tool) and imported into Microsoft Excel V.2016 and Statistical Package for the Social Sciences software V.28.0. Absolute rates per 100 000 population for deaths and DALYs were disaggregated by sex, age group, type of interpersonal violence, SDI and alcohol consumption per capita. In GBD data, the age-standardised deaths and DALYs rates are calculated by adjusting to the global age structure, which is essential for comparing different populations or changes over time. Furthermore, the 95% uncertainty intervals (UIs) were delineated by the 2.5th and 97.5th percentiles, representing the 25th and 975th values, respectively, from the ordered set of 1000 estimates. The 95% UIs were propagated throughout all stages of modelling by creating 1000 values for each death and DALY estimate and performing aggregations across causes and locations at the level of each of the 1000 values for all intermediate steps in the calculation. Trends over time comprised the rates between 1990 and 2019. Additionally, we reported the pace of change between two predefined periods, 2009 vs 2019. The correlation between drivers of violence at the country level and interpersonal violence to children, including country SDI and alcohol consumption per capita, was assessed using Spearman’s rank correlation coefficient.

## Results

**Figure 1:**
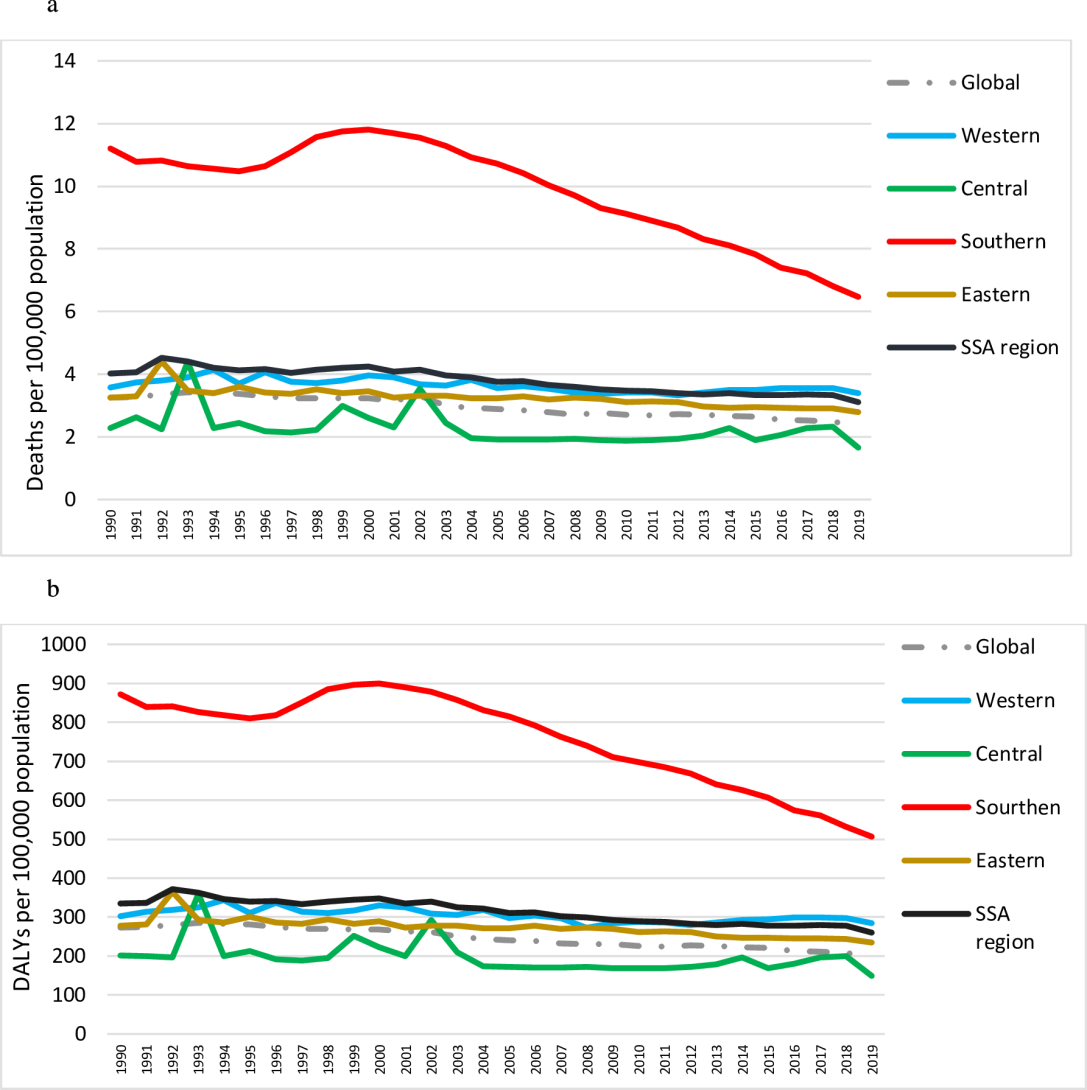
Trend of interpersonal violence injury (a) deaths and (b) disability-adjusted life-years (DALYs) in children<20 years, global and four subregions of sub-Saharan Africa (SSA).

Figure 1 displays the annual trend of interpersonal violence-related deaths and DALYs by subregion. Similar to the global trend, deaths and DALYs in SSA children also declined from 4.0 (95% UI: 3.3 to 4.9) to 3.1 (95% UI: 2.3 to 3.9) and 334.9 (95% UI: 276.4 to 407.7) and 260.3 (95% UI: 197.9 to 321.9) per 100 000 population, respectively, between 1990 and 2019. However, the estimates remained above the global average of 2.4 (95% UI: 2.07 to 2.69) and 199.6 (95% UI: 175.8 to 225.1) deaths and DALYs per 100 000 population, respectively. Minimal downward trends were found over time for the Western, Central and Eastern subregions and a remarkable decrease for Southern SSA, although it remained consistently more affected than all other subregions. Death rates decreased from 11.8 (95% UI: 10.7 to 13.0) to 6.5 (95% UI: 5.2 to 7.9) deaths by 100 000 population and from 899.6 (95% UI: 817.9 to 986.5) to 506.5 (95% UI: 414.9 to 611.1) for DALYs; a 44.9% and 43.7% reduction, respectively. In 2019, Southern SSA had five times the death and DALYs rate of Central SSA, the region least at risk throughout the time period. Central SSA had relatively low death and DALYs rates compared with the global and SSA average rate over the study period.

**Figure 2:**
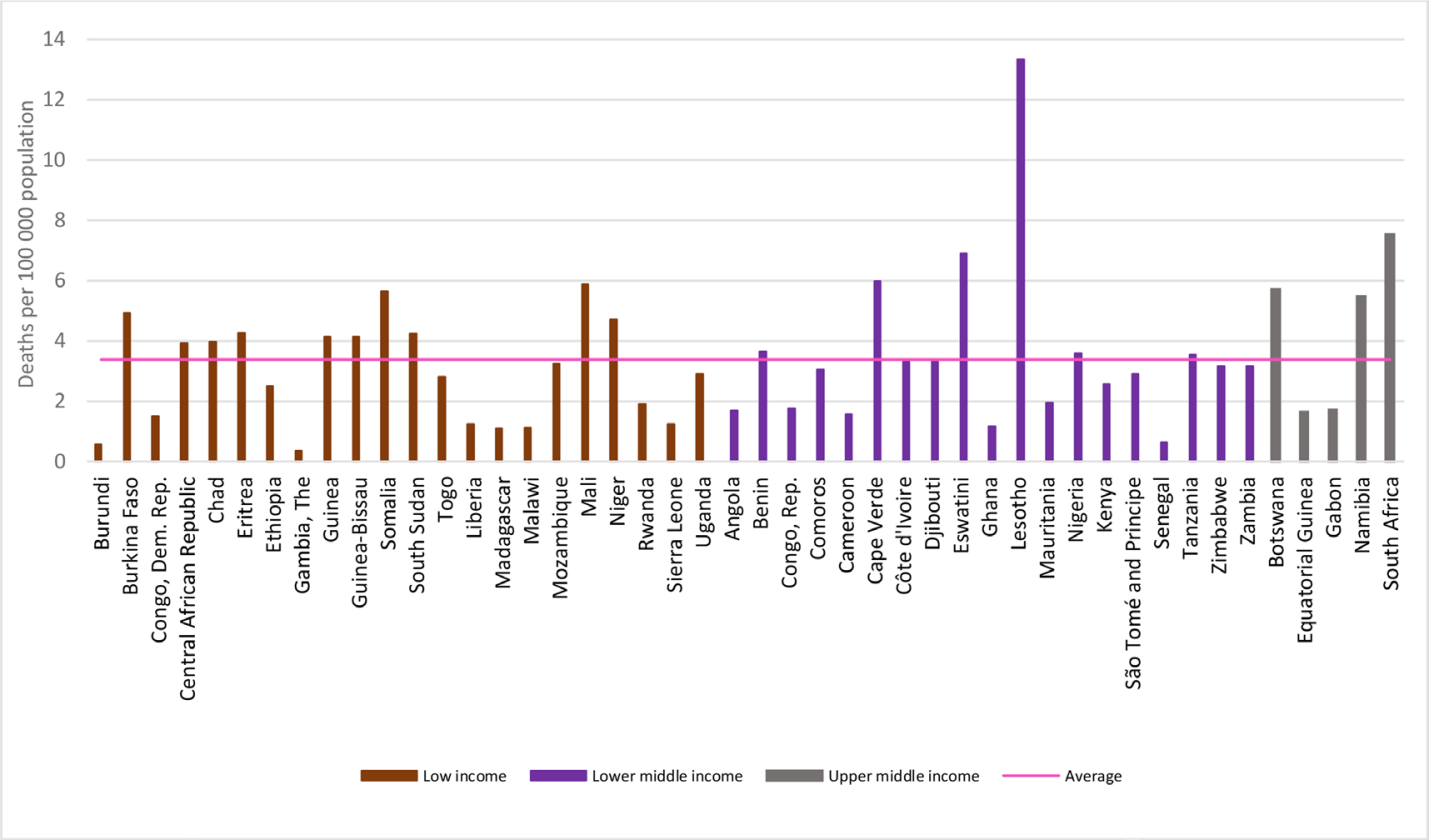
Interpersonal violence deaths in children<20 years by income level and sub-Saharan Africa country, 2019.

Figure 2 presents children’s death rates due to interpersonal violence by country income level in 2019. In total, 10 of the 22 low-income countries had a death rate above the average (3.4 deaths per 100 000) for the region and 6 of the 20 lower-middle-income countries, including 2 countries from Southern SSA with the highest ones for the entire region (Lesotho: 13.3 deaths per 100 000; and Eswatini: 6.9 deaths per 100,000). Among the few upper-middle income countries, three had rates above average and all from Southern SSA (South Africa: 7.5 deaths/100 000; Botswana: 5.7 deaths/100 000; Namibia: 5.5 deaths/100 000).

**Figure 3:**
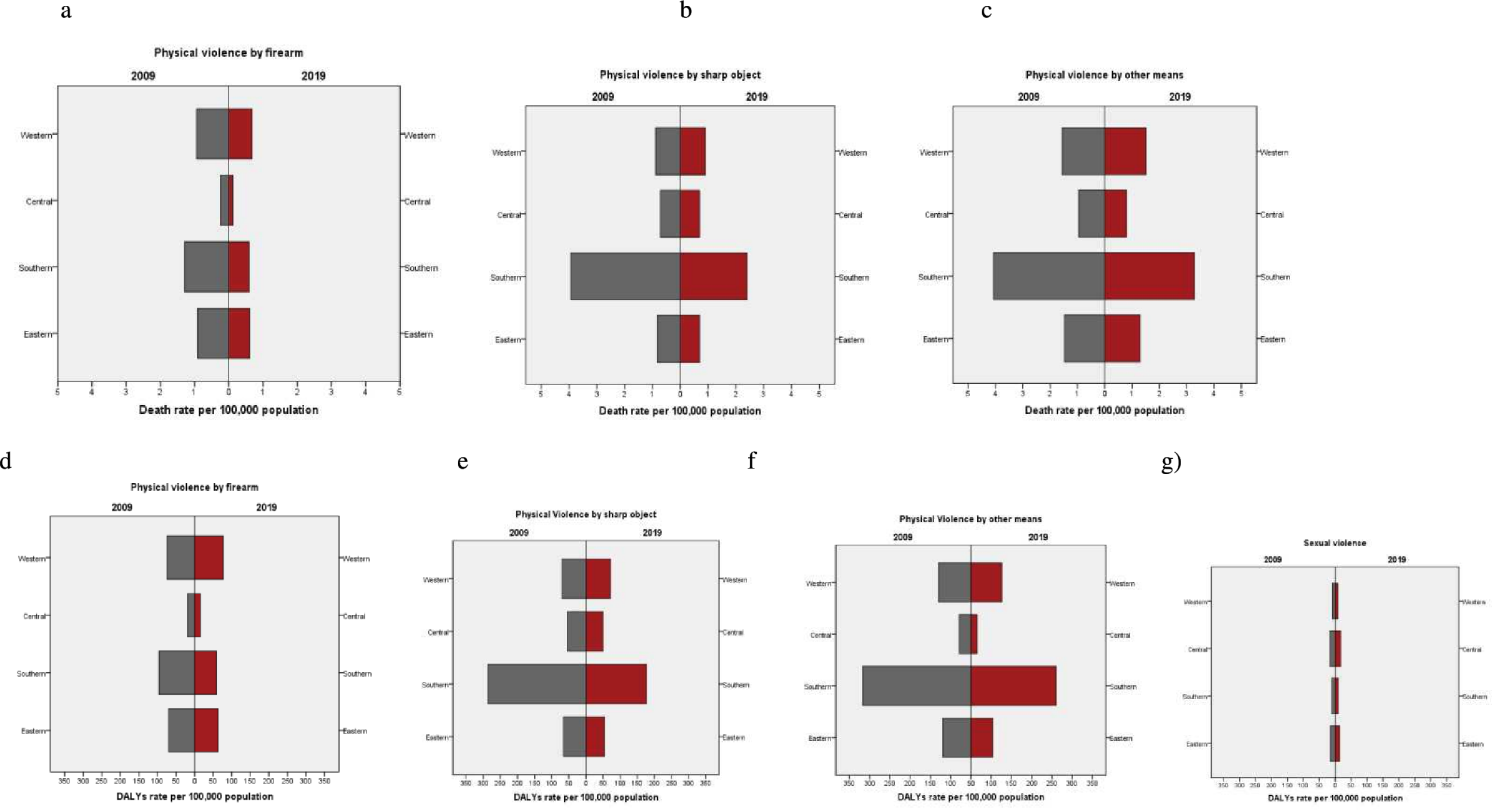
Interpersonal violence rates of (a–c) deaths and (d–g) disability-adjusted life-years (DALYs) in children <20 years by type of violence and sub-region of sub-Saharan Africa, 2009 vs 2019.

Turning to the type of interpersonal violence committed against children, figure 3 shows the rates of deaths and DALYs by subregion in 2009 and 2019 (see online supplemental figure S1–S3). For DALYs, the Southern region dominated again for both reference year and each type of violence, except for sexual violence. The differences between subregions were larger in 2009 than 2019 and more striking for physical violence by sharp object and other means.

Rates in Central SSA surpassed the other regions for sexual violence (17.3 (95% UI: 11.0 to 24.9) in 2009 and 17.6 (95% UI: 10.9 to 26.0) in 2019). The levels were relatively lower compared with the other types of violence and were similar in Eastern SSA for sharp objects (65.9 (95% UI: 47.9 to 83.1) in 2009 and 53.6 (95% UI: 34.3 to 71.5) in 2019). Western and Eastern SSA had similar levels of DALYs across types of violence, except for sexual violence (8.7 (95% UI: 5.5 to 12.7) in 2009 and 9.0 (95% UI: 5.5 to 13.2) in 2019 in Western SSA compared with 15.3 (95% UI: 9.6 to 22.3) in 2009 and 14.5 (95% UI: 9.1 to 21.2) in Eastern SSA in 2019).

Table 1 presents the deaths and DALYs from interpersonal violence-related injuries in 2009 and 2019 by SSA subregions. Except for firearms in 2019, Southern SSA had higher death rates than the other regions for all three forms of violence, but most remarkably for the use of sharp objects (3.9 (95% UI: 2.8 to 4.5) in 2009 and 2.4 (95% UI: 1.4 to 3.1) in 2019) and that of ‘other means’ (4.1 (95% UI: 3.4 to 5.0) in 2009 and 3.3 (95% UI: 2.6 to 4.0) in 2019). UIs were, however, often overlapping.

**Table 1:** Interpersonal violence by region and type of child interpersonal violence, 2009 vs 2019

		Deaths, 95% UI			
		**Physical violence**			**Sexual violence**
		**By firearm**	**By sharp object**	**By other means**	
Southern	2009	1.29 (1.06 to 1.73)	3.94 (2.83 to 4.51)	4.07 (3.44 to 4.96)	
	2019	0.78 (0.59 to 1.06)	2.41 (1.44 to 3.13)	3.28 (2.61 to 4.03)	
Central	2009	0.24 (0.16 to 0.41)	0.71 (0.34 to 0.98)	0.95 (0.69 to 1.39)	
	2019	0.21 (0.12 to 0.36)	0.65 (0.26 to 1.02)	0.80 (0.53 to 1.15)	
Eastern	2009	0.91 (0.74 to 1.10)	0.83 (0.60 to 1.06)	1.48 (1.21 to 1.75)	
	2019	0.82 (0.61 to 1.05)	0.68 (0.43 to 0.90)	1.29 (0.94 to 1.66)	
Western	2009	0.94 (0.72 to 1.15)	0.88 (0.62 to 1.13)	1.56 (1.21 to 1.90)	
	2019	0.97 (0.68 to 1.28)	0.91 (0.53 to 1.25)	1.51 (1.07 to 2.00)	
**DALYs,95%UI**					
Southern	2009	95.83 (79.27 to 127.23)	286.26 (205.97 to 328.03)	317.08 (269.21 to 381.93)	10.91 (6.85 to 15.83)
	2019	58.97 (45.11 to 79.51)	176.81 (106.67 to 228.61)	260.60 (208.76 to 318.44)	10.15 (6.38 to 14.95)
Central	2009	18.77 (12.30 to 32.01)	54.26 (26.22 to 76.14)	78.83 (58.40 to 112.68)	17.28 (11.02 to 24.89)
	2019	16.03 (9.54 to 26.78)	49.77 (20.66 to 77.43)	65.82 (44.13 to 93.87)	17.62 (10.89 to 26.02)
Eastern	2009	70.39 (56.86 to 84.94)	65.84 (47.92 to 83.10)	118.97 (97.73 to 141.03)	15.33 (9.63 to 221.34)
	2019	62.92 (46.39 to 80.98)	53.57 (34.30 to 71.49)	103.91 (75.32 to 132.87)	14.45 (9.12 to 21.16)
Western	2009	74.88 (58.07 to 91.97)	70.41 (50.10 to 89.27)	130.11 (103.08 to 158.55)	8.69 (5.41 to 12.66)
	2019	77.23 (53.69 to 102.24)	72.24 (43.06 to 99.34)	126.13 (90.60 to 166.29)	9.01 (5.51 to 13.24)

DALY, disability-adjusted life-year; UI, uncertainty interval.

**Figure 4:**
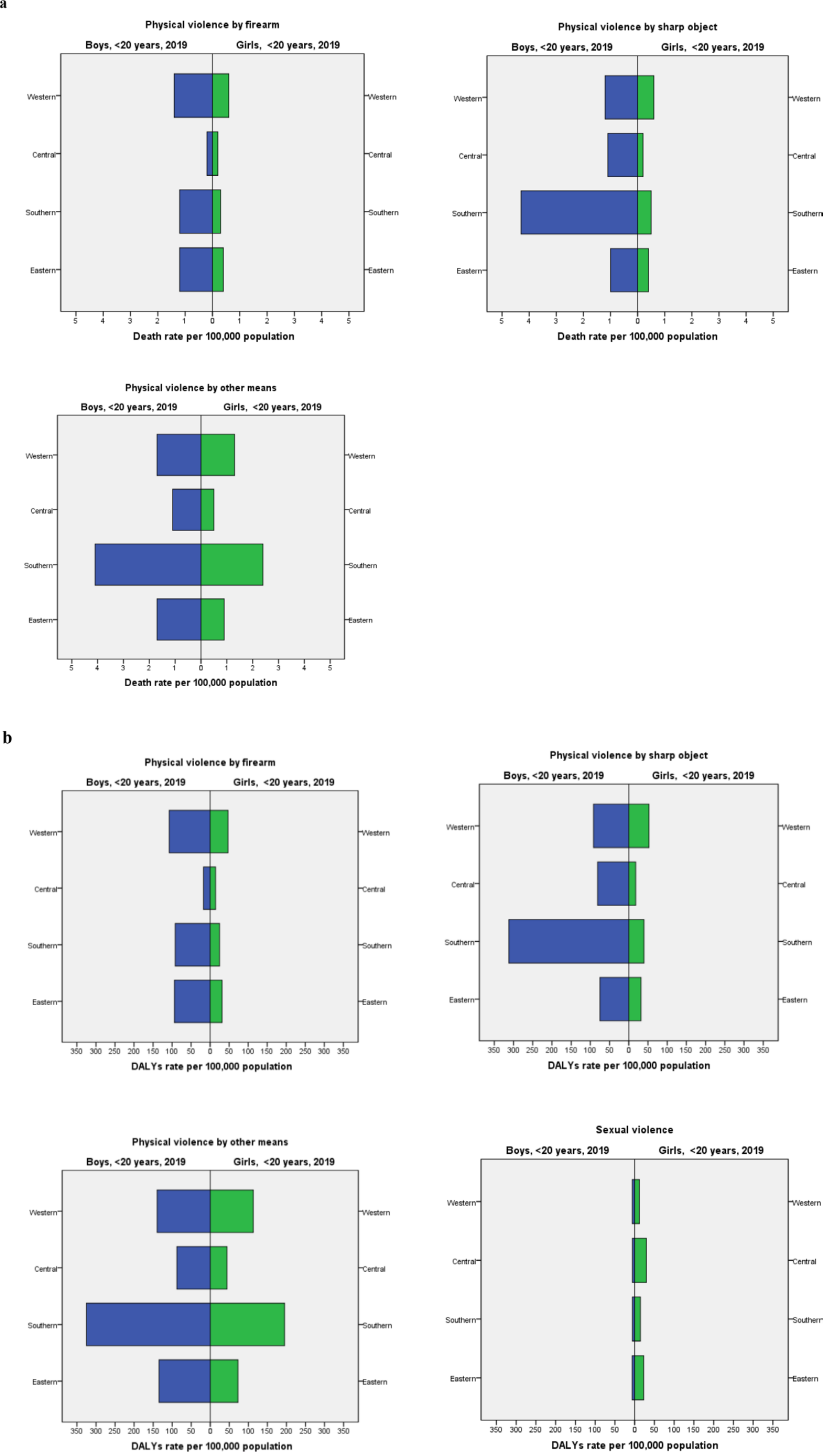
Interpersonal violence: (a) deaths and (b) disability-adjusted life-years (DALYs) in children <20 years in sub-Saharan Africa stratified by sex and region, 2019.

Figure 4 shows child interpersonal violence deaths by sex, type of violence and SSA subregion in 2019. Death rates were almost always much more elevated among boys than girls in all regions except for physical violence by firearm in Central SSA where the rates for both sexes are the lowest and quite comparable (0.2 (95% UI: 0.1 to 0.5) in boys and 0.2 (95% UI: 0.1 to 0.3) in girls). Death by physical violence by other means, the most common type of violence, was far more frequent in Southern Africa for both boys and girls (4.1 (95% UI: 5.4 to 3.1) and 2.4 (95% UI: 1.9 to 3.0), respectively). Our results also show that boys were more victims of death and DALYs due to physical violence at the age of 15–19 years compared with girls (11.7 (95% UI: 7.0 to 16.6) in boys and 2.2 (95% UI: 2.6 to 1.4) in girls for death and 843.7 (95% UI: 1250.5 to 509.4) in boys and 170.5 (95% UI: 220.8 to 101.6) in girls for DALYs). In contrast, girls suffered more morbidity because of sexual violence in the same age group (49.0 (95% UI: 66.1 to 33.6) in girls and 15.1 (95% UI: 20.1 to 10.2) in boys) (online supplemental figures 2 and 3, online supplemental table 5).

**Figure 5:**
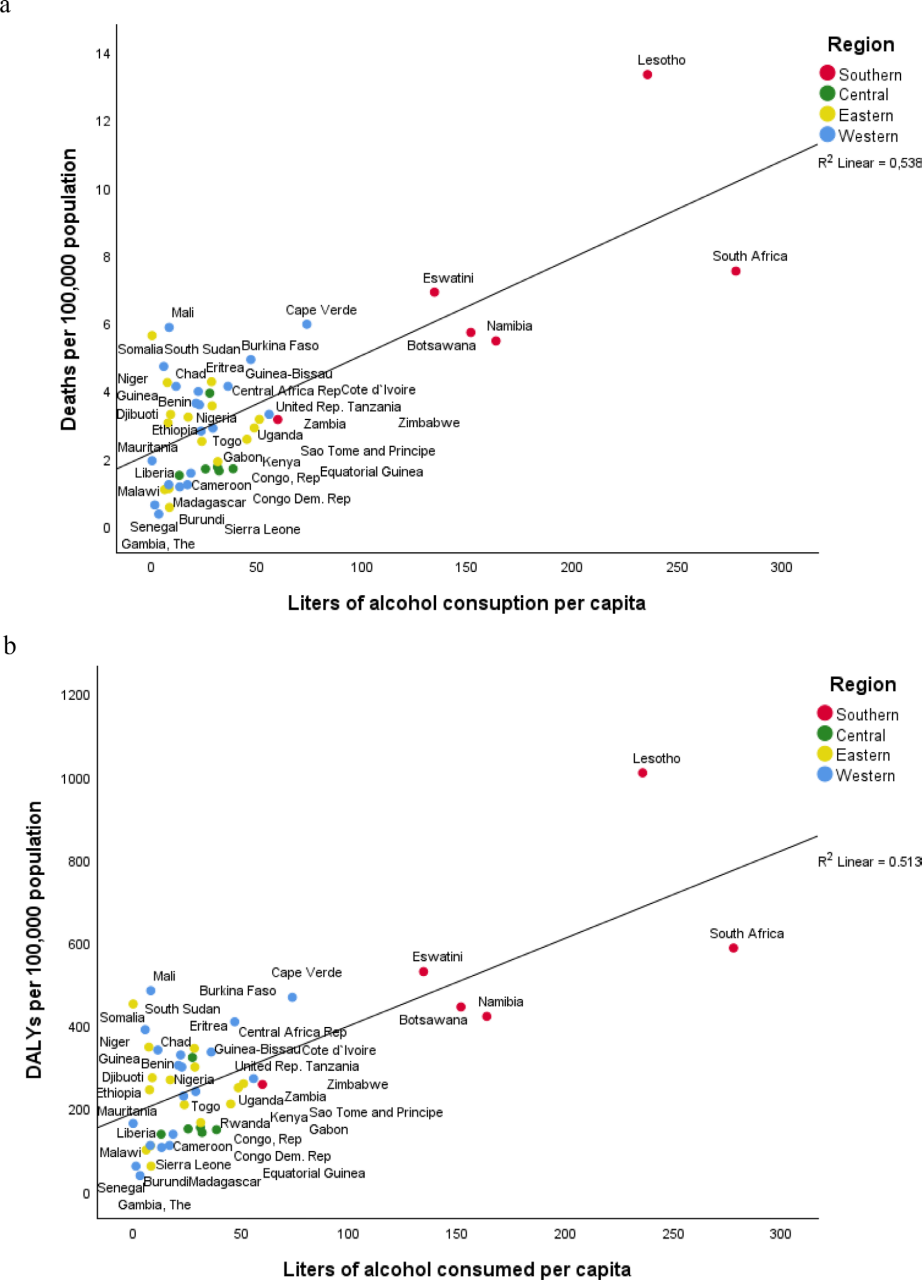
Association between alcohol use and child interpersonal violence (a) deaths and (b) disability-adjusted life-years (DALYs) in children <20 years by sub-Saharan African country and subregion, 2019.

A similar pattern for the three forms of physical violence emerges when looking at DALYs (see figure 5), but there we can see that sexual violence morbidity is consistently higher among girls than boys in all four subregions, predominantly so in Central (29.7 (95% UI: 43.9 to 184) compared with 5.7 (95% UI: 3.5 to 8.4), respectively) and Eastern SSA (23.0 (95% UI: 14.5 to 33.5) compared with 6.1 (95% UI: 3.7 to 9.0), respectively).

Furthermore, there was a non-significant correlation between SDI and child interpersonal violence deaths (r=−0.008, p value=0.958) or DALYs (r=−0.026, p value=0.864) in SSA in 2019 (online supplemental figure S4). A significant correlation was found between alcohol use and child interpersonal violence in SSA in 2019 (figure 5) (values provided in online supplemental table S6). The global and the SSA regional average of alcohol use were similar (37.1 (95% UI: 21.0 to 54.2) and 37.9 (95% UI: 20.2 to 58.1), respectively). When comparing between SSA subregions, Southern SSA had a higher rate of alcohol use (225.5 (95% UI: 128.4 to 329.4)) among the three subregions. A significant correlation was found between alcohol use and child interpersonal violence deaths (r=0.446, p<0.001) and DALYs (r=0.435, p<0.001) in SSA, and this remained significant even when excluding Lesotho and South Africa for both deaths (r=0.367, p<0.001) and DALYs (r=0.358, p<0.001) (see online supplemental figure S5).

## Discussion

### Main findings

As expected from child violence data on previous and shorter time periods,^20^ this study shows decreasing rates of child interpersonal violence deaths and DALYs in the SSA region over the past 30 years, yet the regional level is consistently surpassing the global.^2 17 18^ The literature suggests that the improvements in child interpersonal violence rates over time have been related to the introduction of legal and policy reforms addressing child protection and child violence prevention^20^; intervention-based programmes on violence against children sensitisation, awareness-raising and social norms change^26^; and the intensification of the implementation of the African Charter on the Rights and Welfare of the Child aligned with the Convention on the Rights of the Child.^27^

Also expected is that Southern SSA, by far the most at-risk SSA subregion, heavily influenced this trend during the past two decades.^28^ At the end of the observation period in 2019, death rates varied considerably between SSA countries, and they did not show a significant correlation with country income level. To the contrary, three neighbouring upper-middle-income countries are among the countries with the highest death rates (South Africa, Botswana and Namibia) while the three countries with the highest levels of child interpersonal violence are lower-middle-income countries (Lesotho, Eswatini and Cape Verde).

For both deaths and DALYs, the relative distribution of the means of violence used did not change when comparing 2009 and 2019, with the use of sharp objects and firearms reaching together around 50% at both time points. Southern SSA surpassed the other three subregions for death and DALYs attributed to each form of physical violence (sharp object, firearm or other), which is more apparent in 2009 than in 2019 and less apparent for firearms. Sexual violence rates were much lower than those of the three other forms of violence in all regions. Central SSA stands out along with Eastern SSA as the subregions most affected by sexual violence. Deaths and DALYs rates from all forms of physical violence were higher among adolescent’s boys. Girls had a higher estimate in DALYs for sexual violence. Finally, deaths or DALYs due to interpersonal violence in 2019 did not correlate significantly with country SDI but did correlate significantly with alcohol consumed per capita. This correlation does not imply causation, and the differences in drinking patterns between countries and related alcohol policies limit the interpretation of the results due to the multifactorial risk factors involved.^29^

The GBD data presented here are based on uniform estimation methods, and therefore, the rates and differences observed are not easily comparable with previous studies. In the main, systematic reviews previously published are based on a diversity of data sources where specific forms of violence are considered. Nonetheless, these studies also found the highest death rates in Lesotho and Eswatini (Southern SSA countries), and levels of sexual violence highest in the Democratic Republic of Congo (Central SSA country) where one in every five adolescent girls reported having experienced some form of sexual violence.^2^ The trends examined in this study do not include the changes that took place after 2019 during the COVID-19 pandemic period, which warrant investigation.

In our data, neither country income level nor SDI played a role in explaining risk levels. Research shows that for many causes of injury, age-standardised DALYs rates in all ages were shown to decline with increasing SDI.^28^ GBD experts have suggested that the SDI utility may be improved in the future through consideration of additional societal elements, such as inequality in each component. Income inequality within countries may be a risk factor to explore in future studies as it is known to influence rates of homicide and assault in low-income and middle-income countries, and intimate partner violence.^30^ The mechanisms of influence of income inequality are many and include low investment in children’s rights, violence prevention and in the care and protection of victims in social and healthcare services.^31^ Another potential risk factor in the region may be related to legislation in child protection being poorly enforced, with notable gaps between law and practice.^1^ Also, cultural beliefs, practices and attitudes that endorse gender-based violence may also contribute to norms that legitimise violence against children.^32^ For example, the median percentage of adults who reported that physical violence is necessary to raise and educate children is 39% in SSA as a whole, and ranged from 22% in Congo to 82% in Eswatini.^33^ An earlier global study shows that severe physical punishment of children is almost five times more common in Western SSA than in a range of countries in Eastern Europe, the Balkans and Central Asia, and 40% more common than in a range of countries in the Caribbean, the Middle East and Southeast Asia. Country-based studies on the childhood prevalence of violence in some SSA countries indicated that boys are more likely to be victims of physical violence and harmful practices at home or in the wider community.^34 35^ In contrast, girls are more at risk of sexual violence, mainly at home.^36^ Sexual violence is a form of gender-based violence that predominantly affects girls and is difficult to capture its burden due to high levels of underreporting. Also, large proportions of male victims of sexual violence do not disclose due to stigma, shame and fear of being labelled homosexual.

In addition to the consequences of childhood interpersonal violence on physical and emotional health, experiences of childhood adversity and trauma are also associated with a higher risk for victimisation and perpetration throughout the life course.^37^ There are other well-documented factors that foster violent behaviour detrimental to child safety, among which are alcohol consumption and access to guns.^9 14^ South Africa and Lesotho, two countries with the highest levels of physical violence against children, are countries with the highest alcohol consumption per capita in the world.^38^ The growth of armed conflicts^39^ and the presence of illicit arms market in some countries from Western (eg, Burkina Faso, Nigeria and Chad) and Eastern SSA (eg, Eritrea, Somalia and Djibouti) are very likely to contribute to higher levels of violence against men and women and among boys and girls. Those children may also fall victim to child trafficking, soldiering, forced labour and prostitution.^20^

### Strengths and limitations

Using an ecological framework, this study provides a better understanding of the intraregional distribution of violence-related mortality and morbidity among boys and girls in SSA. It is the most recent and exhaustive description of the like in duration (30 years), geographically (all 46 countries) and outcome based (up to seven indicators of interpersonal violence), and it is informed by harmonised country-based data derived by rigorous compilation procedures.

The analysis has nonetheless some limitations. First, inherent to all register-based studies on violence is that the interpersonal violence levels measured are most likely underestimated due to underreporting by different stakeholders. Second, as it is not possible to assess the level of reporting biases across countries, the differences observed have uncertainty. Therefore, UIs are provided for all results. This applies not only to country or subregion-level comparisons but also to sex-based and age-based stratifications also. Third, the data at hand lack precision with regards to the forms of physical and sexual violence afflicted to children, which may mask differences of importance for the determination of targets for prevention. In addition, GBD are silent about emotional/psychological violence which is both common and coexisting with other types of violence. Fourth, the exclusion of Mauritius and Seychelles has some minor implications for the results of the study. In 2019, these two countries had lower death rates and DALYs than all but one country of SSA (deaths 1.10 per 100 000 for Mauritius and 1.51 for Seychelles; DALYs: 85.39 and 99.50, respectively). Including them in the analysis would have marginally reduced the SSA average and that of the Eastern SSA in which they would be part of according to the World Bank. This, in turn, could have meant slight underestimations of rates and DALYs differences with Southern and Western SSA where these are higher and an overestimation of the differences with Central SSA. Finally, the fact that we have investigated the few available regional-level predictors of violence restricts our ability to explain the influence of factors such as harmful traditional practices, reproductive health factors, the Gini index measuring income inequality,^31^ gender inequality, enforcement of legislation on child protection^40^ and gun control. These are aspects that will be important in future studies.

### Implications for research, practice and policy

Physical violence against children is a pervasive phenomenon in the SSA region, with children at risk in the home, at school and in the wider community. While there has been an encouraging downward trend in violence against children in the SSA region over the past 30 years, comparisons with the global averages or across SSA subregions and countries show that there remains a huge potential for improvement. An important milestone in that direction will be the adoption of child-centric policies at country and regional level that adhere to ground principles embedded in charters and national plans promoting children’s rights, including, education, gender equality and freedom from exploitation. As the SSA region has engaged in documenting the challenges to be faced and progress made thus far,^20^ there are reasons to be optimistic. As indicated in a South African study, a high-risk country from the region, the cost of inaction to the society and economy (5% of the GDP) is unacceptably high.^40^

Regional and international organisations such as the African Partnership to End Violence against Children, the African Child Policy Forum and UNICEF and the WHO have pointed out that lowering rates of violence against children will require multisectoral and long-term actions at different levels, all of which will need to be contextualised. There are encouraging reports of successful evidence-based actions from the seven strategies embedded in the *INSPIRE* framework (implementation and enforcement of laws; norms and values; safe environments; parent and caregiver support; income and economic strengthening; response and support services; and education and life skills)^41^ applied in a variety of African settings.

Alongside those efforts, a broader research agenda is needed to collect indicators that inform on how countries’ political, social, economic and cultural make-up evolve towards child protection and how this, in turn, impacts different forms of violence against boys and girls along the life course. For example, country-wide violence prevention education for children/parents/school personnel/youth-serving organisations, specialised victim services and crisis centres for children, referral and treatment services for offenders to prevent repeat offences.

## Conclusion

The overall trend in SSA is that of declining child interpersonal violence deaths and DALYs. It mirrors the global trends but remains approximately 30% higher for both outcomes in 2019. Variations continue to exist within and between the four subregions, with approximately twice as many deaths and DALYs in Southern SSA versus the SSA average. This detailed assessment of trends between countries over time informs the region on how to identify country-specific and region-wide prevention and health equity measures to advance the implementation of the global 2030 Agenda and Agenda 2063 of the African Union. The significant correlation between alcohol consumed per capita and child interpersonal violence suggests that interventions and policies that focus on reducing population-level consumption of alcohol could also be effective in reducing child interpersonal violence.

Understanding the determinants of the downward trends in SSA driven by remarkable decreases in the southern subregion may pave the way for enhanced child safety protection. Further curbing of disparities between countries and subregions necessitates long-term commitment to evidence-based action plans.

## Supplementary material

10.1136/bmjopen-2023-083070 online supplemental file 1

10.1136/bmjopen-2023-083070 online supplemental file 2

## Data Availability

Data are available in a public, open access repository. Data are available upon reasonable request.
