# Supplementary material for: Trends and demographic differences in interpersonal violence against children in sub-Saharan Africa: findings from the 1990–2019 Global Burden of Disease Study
Source: BMJ Open. 2025 Apr 28;15(4):e083070. doi: 10.1136/bmjopen-2023-083070 (PMC12039018; doi:10.1136/bmjopen-2023-083070)
Supplement: online supplemental file 1 [file bmjopen-15-4-s001.docx]

**Supplemental material**

**Table of Contents (in the order in which they are stated in the manuscript)**

Page 3: Supplementary Table S1 Sub-Saharan African countries by gross national income (GNI), income level classification, SDI/SDI quintile and child interpersonal violence deaths 0-19 years, 2019

Page 5: Supplementary Table S2 Income groups based on gross national income (GNI) per capita and SDI quintiles, 2019

Page 5: Supplementary Table S3 Global Burden of Disease (GBD) interpersonal violence cause, classification, and definition

Page 6: Supplementary Table S4 GATHER checklist of information that should be included in reports of global health estimates, with description of compliance and location information for “Trends and demographic differences in interpersonal violence to children in Sub-Saharan Africa, 1990-2019: findings from the Global Burden of Disease 2019 Study”

Page 9: Supplementary Figure S1 Interpersonal violence deaths (a) and DALYs (b) in children <20 years by type of violence, 2009 vs. 2019 in Sub-Saharan Africa

Page 10: Supplementary Figure S2 Interpersonal violence deaths in children <20 years in Sub-Saharan Africa stratified by sex and age group, 2009 vs. 2019

Page 11: Supplementary Figure S3 Interpersonal violence DALYs in children <20 years in Sub-Saharan Africa stratified by sex and age group, 2009 vs. 2019

Page 12: Supplementary Table S5 Child interpersonal violence by region, sex and type of violence, 2019

Page 13: Supplementary Table S6 Child interpersonal violence deaths and DALYs in Sub-Saharan African countries by alcohol consumption per capita, 2019

Page 16-17: Supplementary Figure S4 Association between Socio-demographic Index (SDI) and interpersonal violence (a) deaths (b) DALYs in children <20 years in Sub-Saharan African countries, 2019

Page 18-19: Supplementary Figure S5 Association between alcohol use and child interpersonal violence (a) deaths and (b) DALYs (in children <20 years by Sub-Saharan African country and sub-region, excluding Lesotho and South Africa 2019

**Supplementary Table S1 Sub-Saharan African countries’ by GNI, income level, SDI/SDI quintile and child interpersonal violence deaths 0-19 years, 2019**

|  | **GNI** | **Income level** | **SDI** | **SDI quintile** | **Interpersonal**  **violence deaths** |
| --- | --- | --- | --- | --- | --- |
| **Southern** | | |  |  | **6.469** |
| Botswana | 7,859.2 | Upper middle | 0.634 | Middle | 5.719 |
| Lesotho | 1,338.5 | Lower middle | 0.507 | Low-middle | 13.329 |
| Namibia | 5,842.1 | Upper middle | 0.612 | Middle | 5.470 |
| South Africa | 6,100.4 | Upper middle | 0.678 | Middle | 7.530 |
| Eswatini | 4.176.8 | Lower middle | 0.577 | Low-middle | 6.908 |
| Zimbabwe | 860.0 | Lower middle | 0.476 | Low-middle | 3.151 |
| **Central** | | |  |  | **1.655** |
| Angola | 3,038.0 | Lower middle | 0.470 | Low-middle | 1.692 |
| Central African Republic | 405.0 | Low income | 0.274 | Low | 3.927 |
| Congo, Rep. | 2,534.5 | Lower middle | 0.568 | Low-middle | 1.751 |
| Congo, Dem. Rep. | 500.6 | Low income | 0.382 | Low | 1.501 |
| Equatorial Guinea | 8,927.4 | Upper middle | 0.685 | Middle | 1.634 |
| Gabon | 8,112.3 | Upper middle | 0.656 | Middle | 1.698 |
| **Eastern** | | |  |  | **2.790** |
| Burundi | 309.9 | Low | 0.284 | Low | 0.557 |
| Comoros | 1,349.8 | Lower middle | 0.455 | Low-middle | 3.049 |
| Djibouti | 2,936.2 | Lower middle | 0.459 | Low-middle | 3.301 |
| Eritrea | 342.6 | Low | 0.396 | Low | 4.265 |
| Ethiopia | 953.2 | Low | 0.343 | Low | 2.504 |
| Kenya | 1,997.6 | Lower middle | 0.508 | Low-middle | 2.564 |
| Madagascar | 463.9 | Low | 0.396 | Low | 1.083 |
| Malawi | 370.7 | Low | 0.384 | Low | 1.113 |
| Mozambique | 484.4 | Low | 0.307 | Low | 3.221 |
| Rwanda | 770.1 | Low | 0.429 | Low | 1.908 |
| Somalia | 410.0 | Low | 0.081 | Low | 5.627 |
| United Rep. of Tanzania | 1,104.8 | Lower middle | 0.423 | Low | 3.553 |
| Uganda | 770.1 | Low | 0.404 | Low | 2.902 |
| Zambia | 1,307.0 | Lower middle | 0.505 | Low-middle | 3.156 |
| South Sudan | 275.2 | Low | 0.363 | Low | 4.242 |
| **Western** | | |  |  | **3.394** |
| Benin | 1,216.6 | Lower middle | 0.352 | Low | 3.628 |
| Burkina Faso | 718.0 | Low | 0.257 | Low | 4.918 |
| Cameroon | 1,514.6 | Lower middle | 0.490 | Low-middle | 1.567 |
| Cape Verde | 3,598.6 | Lower middle | 0.525 | Low-middle | 5.960 |
| Chad | 861.2 | Low | 0.238 | Low | 3.977 |
| Côte d'Ivoire | 1,691.3 | Lower middle | 0.408 | Low | 3.304 |
| Gambia, The | 755.0 | Low | 0.399 | Low | 0.358 |
| Ghana | 2,223.4 | Lower middle | 0.557 | Low-middle | 1.163 |
| Guinea | 981.0 | Low | 0.325 | Low | 4.129 |
| Guinea-Bissau | 786.3 | Low | 0.355 | Low | 4.130 |
| Liberia | 703.8 | Low | 0.370 | Low | 1.227 |
| Mali | 924.2 | Low | 0.263 | Low | 5.867 |
| Mauritania | 1,392.5 | Lower middle | 0.496 | Low-middle | 1.935 |
| Niger | 405.1 | Low | 0.162 | Low | 4.716 |
| Nigeria | 2,222.0 | Lower middle | 0.515 | Low-middle | 3.584 |
| São Tomé and Principe | 1,691.3 | Lower middle | 0.502 | Low-middle | 2.895 |
| Senegal | 1,441.4 | Lower middle | 0.389 | Low | 0.629 |
| Sierra Leone | 546.6 | Low | 0.347 | Low | 1.227 |
| Togo | 671.4 | Low | 0.417 | Low | 2.807 |

GNI: gross national income; SDI: Socio-demographic Index

**Supplementary Table S2. Income groups based on gross national income (GNI) per capita and socio-demographic index (SDI) quintiles, 2019**

|  | **Lower bounds** | **Upper bound** |
| --- | --- | --- |
| **GNI** |  |  |
| Low | < $1,045 | $1,045 |
| Lower-middle | $1,046 | 4,095 |
| Upper-middle | $4,096 to | 12,695 |
| High | $12,696 | >$12,696 |
| **SDI quintiles** |  |  |
| Low | 0 | 0.454743 |
| Low-middle | 0.454743 | 0.607679 |
| Middle | 0.607679 | 0.689504 |
| High-middle | 0.689504 | 0.805129 |
| High | 0.805129 | 1 |

**Supplementary Table S3: Global Burden of Disease (GBD) interpersonal violence cause, classification, and definition**

| **Cause** | **Classification** | **Definition** |
| --- | --- | --- |
| Interpersonal violence | Level 3 | ﻿Death or disability from intentional use of physical force or power, threatened or actual, from another person or group not including military or police force |
| Physical violence by firearm | Level 4 | ﻿Death or disability from intentional use of a firearm by another person, not including military or police forces |
| Physical violence by sharp object | Level 4 | ﻿Death or disability from intentional use of physical force or power by a sharp object from another person, not including military or police forces |
| Physical violence by other means | Level 4 | ﻿Death or disability from intentional use of physical force or power by an object other than a firearm or sharp object from another person not including legal, military, or police forces |
| Sexual violence | Level 4 | ﻿Experiencing at least one event of sexual violence in the last year, where sexual violence is any sexual assault, including rape as well as other forms of assault such as unwanted sexual touching |

**Supplementary Table S4 GATHER checklist of information that should be included in reports of global health estimates, with description of compliance and location information for “****Trends and demographic differences in interpersonal violence to children in Sub-Saharan Africa, 1990-2019: findings from the Global Burden of Disease 2019 Study”**

| **Item**  **#** | **GATHER checklist item** | **Description of compliance** | **Reference** |
| --- | --- | --- | --- |
| Objectives and funding | | |  |
| 1 | Define the indicator(s), populations (including age, sex, and geographic entities), and time period(s) for which estimates were made | Narrative provided in paper and  appendix describing indicators, definitions,  and populations | Main text (Methods) and supplementary files |
| 2 | List the funding sources for the work | Funding sources listed in paper-Contributions section | Section funding |
| **Data Inputs** | | |  |
| *For all data inputs from multiple sources that are synthesized as part of the study:* | | |  |
| 3 | Describe how the data were identified and how the data were accessed | Narrative description of data seeking methods provided | Main text (Methods) and supplementary files |
| 4 | Specify the inclusion and exclusion criteria. Identify all ad-hoc exclusions | An interactive, online data source tool that  provides metadata for data sources by  component, geography, cause, risk, or  impairment has been developed | Online data citation tool:  https://ghdx.healthdata.org/gbd-2019 |
| 5 | Provide information on all included data sources and their main characteristics. For each data source used, report reference information or contact name/institution, population  represented, data collection method, year(s) of data collection, sex and age range, diagnostic criteria or measurement method, and sample size, as relevant | An interactive, online data source tool that  provides metadata for data sources by  component, geography, cause, risk, or  impairment has been developed | Online data citation tool:  https://ghdx.healthdata.org/gbd-2019 |
| 6 | Identify and describe any categories of input data that have potentially important biases  (e.g., based on characteristics listed in item 5) | Summary of known  biases by cause included in online data source tool | Online at Global Health Data Exchange (GHDx,  http://ghdx.healthdata.org/) |
| *For data inputs that contribute to the analysis but were not synthesized as part of the study:* | | |  |
| 7 | Describe and give sources for any other data inputs | Online at  https://ghdx.healthdata.org/gbd  -2019 | Online at Global Health Data Exchange (GHDx,  http://ghdx.healthdata.org/) |
| *For all data inputs:* | | |  |
| 8 | Provide all data inputs in a file format from which data can be efficiently extracted (e.g., a spreadsheet rather than a PDF), including all relevant meta-data listed in item 5. For any  data inputs that cannot be shared because of ethical or legal reasons, such as third-party ownership, provide a contact name or the name of the institution that retains the right to the data | Downloads of input data available through online tools, including data visualisation tools and data query tools; input data not available in  tools will be made available upon request | Online data  visualisation tools,  data query tools, and  the Global Health Data  Exchange |
| **Data analysis** | | |  |
| 9 | Provide a conceptual overview of the data analysis method. A diagram may be helpful. | Main text (Methods),  Supplementary files | https://ghdx.healthdata.org/gbd  -2019 |
| 10 | Provide a detailed description of all steps of the analysis, including mathematical formulae. This description should cover, as relevant, data cleaning, data pre-processing, data adjustments and weighting of data sources, and mathematical or statistical model(s). | Provided in the GBD 2019 Methods appendices,  https://ghdx.healthdata.org/gbd  -2019 | GBD  2019 Methods appendices,  https://ghdx.healthdata.org/gbd  -2019 |
| 11 | Describe how candidate models were evaluated and how the final model(s) were selected. | Provided in the GBD 2019 Methods appendices,  https://ghdx.healthdata.org/gbd  -2019 | GBD  2019 Methods appendices,  https://ghdx.healthdata.org/gbd  -2019 |
| 12 | Provide the results of an evaluation of model performance, if done, as well as the results of any relevant sensitivity analysis. | Provided in the GBD 2019 Methods appendices,  https://ghdx.healthdata.org/gbd  -2019 | GBD  2019 Methods appendices,  https://ghdx.healthdata.org/gbd  -2019 |
| 13 | Describe methods for calculating uncertainty of the estimates. State which sources of uncertainty were, and were not, accounted for in the uncertainty analysis. | Provided in the GBD 2019 Methods appendices,  https://ghdx.healthdata.org/gbd  -2019 | GBD  2019 Methods appendices,  https://ghdx.healthdata.org/gbd  -2019 |
| 14 | State how analytic or statistical source code used to generate estimates can be accessed. | Provided in the GBD 2019 Methods appendices,  https://ghdx.healthdata.org/gbd  -2019 | GBD  2019 Methods appendices,  https://ghdx.healthdata.org/gbd  -2019 |
| **Results and Discussion** | | |  |
| 15 | Provide published estimates in a file format from which data can be efficiently extracted. | Results are available upon publication  online through data visualization tools, the  Global Health Data Exchange, an online  results query tool | GBD 2019 results are available through online  data visualisation tools, the Global Health Data  Exchange, and the  online data query tool |
| 16 | Report a quantitative measure of the uncertainty of the estimates (e.g., uncertainty intervals). | Uncertainty intervals provided with the results, supplementary files, and online data tools  https://ghdx.healthdata.org/gbd  -2019 | Main text, appendix, and  online data tools (data  visualisation tools, data  query tools, and the  Global Health Data  Exchange) |
| 17 | Interpret results in light of existing evidence. If updating a previous set of estimates, describe the reasons for changes in estimates. | Discussion of  methodological changes  between GBD rounds  provided in the narrative  of the manuscript | Main text (Discussion) |
| 18 | Discuss limitations of the estimates. Include a discussion of any modelling assumptions or data limitations that affect interpretation of the estimates. | Discussion of limitations  provided in the limitations section of the main paper | Main text (Limitations) |

1. Deaths

1. DALYs

**Supplementary figure S1. Interpersonal violence deaths (a) and DALYs (b) in children <20 years by type of violence, 2009 vs. 2019 in Sub-Saharan Africa**

1. Physical violence deaths, boys b) Physical violence deaths, girls

**Supplementary figure S2 Interpersonal violence deaths in children <20 years in Sub-Saharan Africa stratified by sex and age group, 2009 vs. 2019**

a) Physical violence DALYs, boys b) Physical violence DALYs, girls

c) Sexual violence DALYs, boys d) Sexual violence DALYs, girls

**Supplementary figure S3 Interpersonal violence DALYs in children <20 years in Sub-Saharan Africa stratified by sex and age group, 2009 vs. 2019**

**Supplementary table S5 Child interpersonal violence by region, sex and type of violence, 2019**

|  | **Sex** | **Physical violence** | | | **Sexual violence** |
| --- | --- | --- | --- | --- | --- |
| **Deaths** |  | **By firearm** | **By sharp object** | **By other means** |  |
| Southern | Boys | 1.2 (95% UI: 0.9-1.8) | 4.3 (95% UI: 2.4-5.6) | 4.1 (95% UI: 3.1-5.4) |  |
|  | Girls | 0.3 (95% UI: 0.2-0.4) | 0.5 (95% UI: 0.3-0.7) | 2.4 (95% UI: 1.8-3.0) |  |
| Central | Boys | 0.2 (95% UI: 0.1-0.6) | 1.1 (95% UI: 0.3-1.7) | 1.1 (95% UI: 0.6-1.7) |  |
|  | Girls | 0.2 (95% UI: 0.1-0.3) | 0.2 (95% UI: 0.1-0.4) | 0.5 (95% UI: 0.3-0.8) |  |
| Eastern | Boys | 1.2 (95% UI: 0.9-1.6) | 1.0 (95% UI: 0.6-1.3) | 1.7 (95% UI: 1.2-2.2) |  |
|  | Girls | 0.4 (95% UI: 0.3-0.5) | 0.4 (95% UI: 0.2-0.5) | 0.9 (95% UI: 0.6-1.2) |  |
| Western | Boys | 1.4 (95% UI: 0.9-1.8) | 1.2 (95% UI: 0.6-1.7) | 1.7 (95% UI: 1.1-2.2) |  |
|  | Girls | 0.6 (95% UI: 0.4-1.8) | 0.6 (95% UI: 0.4-1.0) | 1.3 (95% UI: 0.9-1.8) |  |
| **DALYs** | | | | | |
| Southern | Boys | 91.7 (95% UI:66.8-133.8) | 312.1 (95% UI:175.9-405.3) | 325.2 (95% UI: 249.2-418.0) | 5.6 (95% UI:3.5-8.4) |
|  | Girls | 43.3 (95% UI:21.5-43.3) | 39.6 (95% UI:26.5-56.1) | 195.0 (95% UI: 154.2-240.7) | 14.7 (95% UI:9.3-21.8) |
| Central | Boys | 17.5 (95% UI:8.5-40.8) | 81.1 (95% UI:29.2-129.8) | 87.5 (95% UI: 53.6-136.2) | 5.7 (95% UI:3.5-8.4) |
|  | Girls | 14.5 (95% UI:7.9-23.6) | 18.2 (95% UI:8.8-34.3) | 44.0 (95% UI: 67.0-26.5) | 29.7 (95% UI:18.4-43.9) |
| Eastern | Boys | 93.8 (95% UI:67.1-122.1) | 74.9 (95% UI:44.7-46.1) | 134.5 (95% UI: 94.7-171.8) | 6.1 (95% UI:3.6.7-9.0) |
|  | Girls | 31.4 (95% UI:21.5-43.4) | 31.8 (95% UI:20.7-46.1) | 72.7 (95% UI: 50.4-98.6) | 23.0 (95% UI:14.5-33.4) |
| Western | Boys | 107.5 (95% UI:72.0-144.9) | 91.8 (95% UI:48.9-130.0) | 139.3 (95% UI: 93.7-190.0) | 6.0 (95% UI:3.7-9.0) |
|  | Girls | 47.0 (95% UI:32.6-64.2) | 52.7 (95% UI:32.9-79.1) | 113.0 (95% UI: 79.3-151.5) | 12.0 (95% UI:7.3-17.6) |

UI: uncertainty interval

**Supplementary table 6 Child interpersonal violence deaths and DALYs in Sub-Saharan African countries by alcohol consumption per capita, 2019**

| **Country name** | **Alcohol per capita value** | **Child interpersonal violence deaths** | **Child interpersonal violence DALYs** |
| --- | --- | --- | --- |
| **Global** | **37.1 (95% UI: 21.0-54.2)** | **2.4 (95% UI: 2.07-2.69)** | **199.6 (95% UI: 175.8-225.1)** |
| **SSA region** | **37.9 (95% UI: 20.2-58.1)** | **3.11 (95% UI: 2.31-3.91)** | **260.3 (95% UI: 197.9-321.9)** |
| **Southern SSA** | **225.5 (95% UI: 128.4-329.4)** | **6.5 (95% UI: 5.2-7.9)** | **506.5 (95% UI: 414-611.9)** |
| Botswana | 151.7 (95% UI: 61.9-262.5) | 5.7 (95% UI: 3.4-8.6) | 445.8 (95% UI: 273.0-657.8) |
| Lesotho | 235.7 (95% UI: 103.6-393.9) | 13.3 (95% UI: 9.1-19.3) | 1009.7 (95% UI: 699.8-1441.6) |
| Namibia | 163.6 (95% UI: 75.4-289.5) | 5.5 (95% UI: 3.31-8.2) | 422.5 (95% UI: 264.0-619.6) |
| South Africa | 277.8 (95% UI: 159.2-407.3) | 7.5 (95% UI: 5.9-9.3) | 587.4 (95% UI: 464.7-720.0) |
| Eswatini | 134.4 (95% UI: 66.1-234.9) | 6.9 (95% UI: 4.4-10.5) | 530.3 (95% UI: 350.3-786.0) |
| Zimbabwe | 59.8 (95% UI: 27.2-105.1) | 3.2 (95% UI: 2.0-4.6) | 258.0 (95% UI: 175.9-363.9) |
| **Central SSA** | **17.7 (95% UI: 6.9-29.7)** | **1.7 (95% UI: 1.1-2.3)** | **149.2 (95% UI: 107.4-202.0)** |
| Angola | 25.4 (95% UI: 12.8-42.6) | 1.7 (95% UI: 0.9-2.6) | 150.6 (95% UI: 91.5-219.0) |
| Central African Republic | 27.4 (95% UI: 6.8-54.9) | 3.9 (95% UI: 2.4-6.1) | 323.1 (95% UI: 208.9-482.4) |
| Congo, Rep. | 31.1 (95% UI: 12.1-54.6) | 1.8 (95% UI: 1.0-2.7) | 153.9 (95% UI: 100.7-228.0) |
| Congo, Dem. Rep. | 12.9 (95% UI: 2.6-24.4) | 1.5 (95% UI: 1.0-2.3) | 138.2 (95% UI: 93.6-196.0) |
| Equatorial Guinea | 31.9 (95% UI: 13.3-61.8) | 1.6 (95% UI: 0.8-3.1) | 142.7 (95% UI: 77.9-246.05) |
| Gabon | 38.6 (95% UI:16.8-67.4) | 1.7 (95% UI: 0.9-2.8) | 149.2 (95% UI: 91.2-229.3) |
| **Eastern SSA** | **26.6 (95% UI: 11.8-42.5)** | **2.8 (95% UI: 2.1-3.5)** | **234.8 (95% UI: 177.2-291.5)** |
| Burundi | 8.3 (95% UI: 4.0-14.5) | 0.6 (95% UI: 0.3-0.9) | 61.4 (95% UI: 37.8-87.2) |
| Comoros | 7.6 (95% UI: 0.6-18.3) | 3.0 (95% UI: 1.5-4.8) | 245.9 (95% UI: 126.0-381.0) |
| Djibouti | 8.8 (95% UI: -2.1-24.2) | 3.3 (95% UI: 1.9-5.3) | 274.4 (95% UI: 160.8-426.3) |
| Eritrea | 28.3 (95% UI: 4.2-62.7) | 4.6 (95% UI: 2.4-7.0) | 345.9 (95% UI: 205.6-538.3) |
| Ethiopia | 23.7 (95% UI: 7.2-41.8) | 2.5 (95% UI: 1.9-3.2) | 209.8 (95% UI: 162.1-269.2) |
| Kenya | 45.1 (95% UI: 19.6-75.9) | 2.6 (95% UI: 2.0-3.3) | 211.7 (95% UI: 168.5-270.5) |
| Madagascar | 6.0 (95% UI: 1.2-12.0) | 1.0 (95% UI: 0.5-5.6) | 100.5 (95% UI: 57.3-146.3) |
| Malawi | 8.0 (95% UI: 3.1-14.9) | 1.1 (95% UI: 0.5-1.9) | 109.9 (95% UI: 59.8-172.7) |
| Mozambique | 17.2 (95% UI: 2.8-37.0) | 3.2 (95% UI: 1.9-4.8) | 268.7 (95% UI: 164.3-394.6) |
| Rwanda | 31.3 (95% UI: 14.6-52.8) | 1.9 (95% UI: 1.2-3.0) | 165.8 (95% UI: 109.8-291.5) |
| Somalia | 0 | 5.6 (95% UI: 3.2-9.8) | 451.8 (95% UI: 265.0-757.9) |
| United Rep. of Tanzania | 28.5 (95% UI: 13.6-14.0) | 3.6 (95% UI: 2.4-5.2) | 300.5 (95% UI: 24.9-434.5) |
| Uganda | 48.6 (95% UI: 24.0-79.6) | 2.9 (95% UI: 1.8-4.4) | 250.4 (95% UI: 164.6-371.4) |
| Zambia | 51.0 (95% UI: 21.4-85.6) | 3.2 (95% UI: 2.1-4.6) | 259.7 (95% UI: 184.1-365.0) |
| South Sudan | 7.3 (95% UI: -1.5-19.3) | 4.2 (95% UI: 2.9-6.2) | 348.2 (95% UI: 241.3-497.3) |
| **Western SSA** | **21.6 (95% UI: 11.8-42.5)** | **3.4 (95% UI: 2.4-4.5)** | **284.6 (95% UI: 201.1-370.5)** |
| Benin | 20.9 (95% UI: 8.5-38.7) | 3.6 (95% UI: 2.2-5.3) | 303.7 (95% UI: 194.0-458.6) |
| Burkina Faso | 47.0 (95% UI: 20.8-78.7) | 4.9 (95% UI: 3.3-7.1) | 409.6 (95% UI: 273.2-593.8) |
| Cameroon | 18.5 (95% UI: 9.3-31.6) | 1.6 (95% UI: 0.9-2.5) | 137.9 (95% UI: 86.2-206.1) |
| Cape Verde | 73.7 (95% UI: 39.4-120.7) | 6.0 (95% UI: 4.0-7.9) | 467.6 (95% UI: 320.6-613.9) |
| Chad | 22.0 (95% UI: 7.6-39.7) | 4.0 (95% UI: 2.6-5.6) | 329.5 (95% UI: 225.7-456.6) |
| Côte d'Ivoire | 55.7 (95% UI: 25.0-95.7) | 3.3 (95% UI: 1.9-5.1) | 272.7 (95% UI: 162.8-413.9) |
| Gambia, The | 3.2 (95% UI: 1.2-5.3) | 0.4 (95% UI: 0.2-0.5) | 38.4 (95% UI: 26.2-54.1) |
| Ghana | 13.2 (95% UI: 5.3-22.9) | 1.2 (95% UI: 0.7-1.8) | 106.2 (95% UI: 69.8-156.9) |
| Guinea | 11.4 (95% UI: 4.0-19.8) | 4.1 (95% UI: 2.7-6.2) | 341.4 (95% UI: 226.3-507.2) |
| Guinea-Bissau | 36.1 (95% UI: 16.2-61.4) | 4.1 (95% UI: 2.7-6.3) | 336.2 (95% UI: 220.2-505.3) |
| Liberia | 16.8 (95% UI: 28.2-8.3) | 1.2 (95% UI: 0.8-1.8) | 111.5 (95% UI: 76.5-152.2) |
| Mali | 8.2 (95% UI: 3.1-14.7) | 5.7 (95% UI: 3.8-5.9) | 484.6 (95% UI: 319.8-703.6) |
| Mauritania | 0 | 1.9 (95% UI: 1.0-3.6) | 164..5 (95% UI: 92.7-257.4) |
| Niger | 5.6 (95% UI: 0.1-13.7) | 4.7 (95% UI: 2.6-7.5) | 390.0 (95% UI: 220.6-610.0) |
| Nigeria | 22.5 (95% UI: 10.7-40.1) | 3.6 (95% UI: 2.3-4.9) | 300.6 (95% UI: 197.1-407.0) |
| São Tomé and Principe | 28.9 (95% UI: 14.9-48.0) | 2.9 (95% UI: 1.7-4.5) | 241.9 (95% UI: 150.1-372.4) |
| Senegal | 1.3 (95% UI: 0.6-2.3) | 0.6 (95% UI: 0.4-1.0) | 61.6 (95% UI: 39.4-92.0) |
| Sierra Leone | 7.9 (95% UI: 3.7-13.3) | 1.2 (95% UI: 0.7-2.0) | 1111.5 (95% UI: 63.2-173.1) |
| Togo | 23.3 (95% UI: 10.1-40.7) | 2.8 (95% UI: 1.8-4.3) | 230.8 (95% UI: 151.0-340.6) |

UI: uncertainty interval

1. SDI and deaths


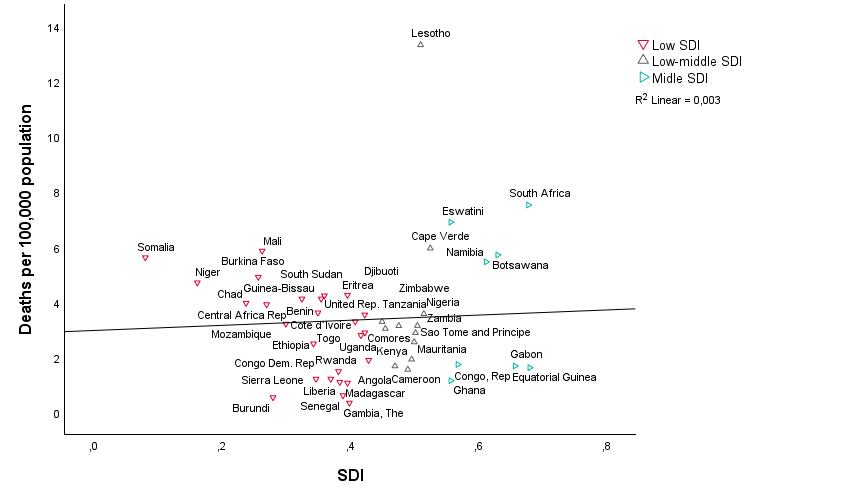


b) SDI and DALYs


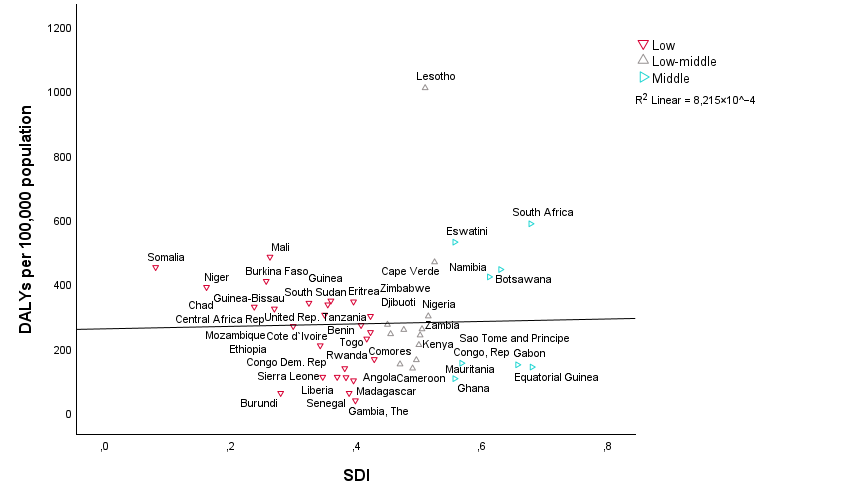


**Supplementary figure S4 Association between Socio-demographic Index (SDI) and interpersonal violence deaths (a) deaths and (b) DALYs in children <20 years in Sub-Saharan African countries, 2019**

a)


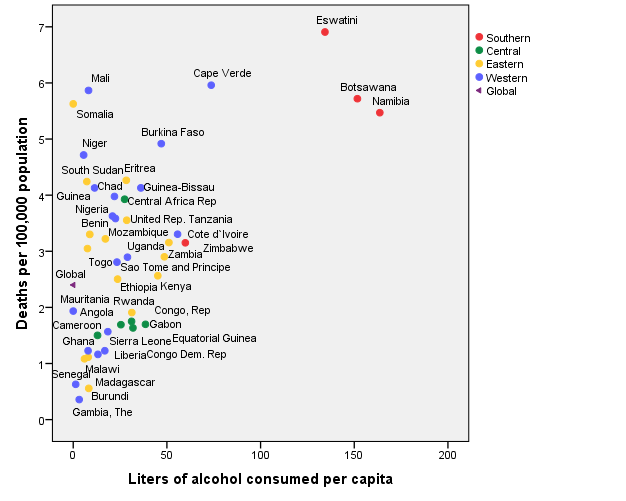


b)


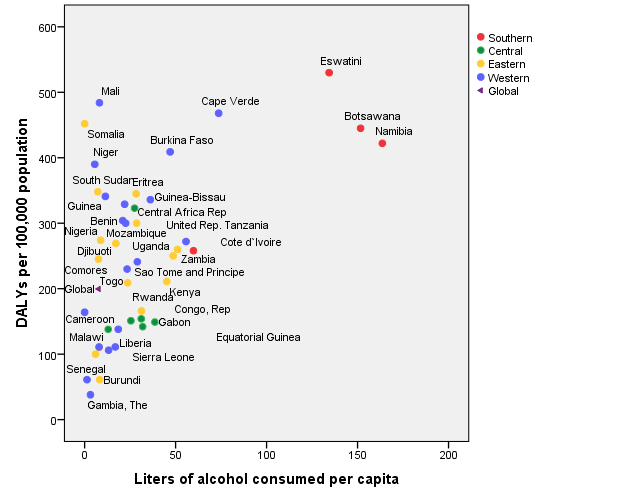


**Supplementary figure S5 Association between alcohol use and child interpersonal violence (a) death (b) DALYs (in children <20 years by Sub-Saharan African country and sub-region, excluding Lesotho and South Africa**
