# Supplementary material for: Trends and demographic differences in interpersonal violence against children in sub-Saharan Africa: findings from the 1990–2019 Global Burden of Disease Study
Source: BMJ Open. 2025 Apr 28;15(4):e083070. doi: 10.1136/bmjopen-2023-083070 (PMC12039018; doi:10.1136/bmjopen-2023-083070)
Supplement: online supplemental file 2 [file bmjopen-15-4-s002.docx]

**GBD 2019 SSA Child Interpersonal Violence Collaborators**

Sergio Keita Nhassengo,^1,2^ Yohannes Habtegiorgis Abate,^3^ Mesfin Abebe,^4^ Kedir Hussein Abegaz,^5^ Prof Olumide Abiodun,^6^ Richard Gyan Aboagye,^7^ Kenneth Setorwu Adde,^8^ Oluwafemi Atanda Adeagbo,^9,10^ Abiola Victor Victor Adepoju,^11,12^ Muktar Beshir Ahmed,^13,14^ Timothy Olukunle Aladelusi,^15,16^ Deborah Oyine Aluh,^17,18^ Hubert Amu,^19^ Idowu Thomas Aruleba,^20^ Getnet Melaku Ayele,^4^ Amadou Barrow,^21,22^ Hameed Akande Bashiru,^23^ Obasanjo Afolabi Bolarinwa,^24^ Malizgani Paul Chavula,^25,26^ Berihun Assefa Dachew,^27,28^ Berecha Hundessa Demessa,^29^ Isaac Oluwafemi Dipeolu,^30^ Robert Kokou Dowou,^31^ Michael Ekholuenetale,^32^ Temitope Cyrus Ekundayo,^33^ Adewale Oluwaseun Fadaka,^34,35^ Adeniyi Francis Fagbamigbe,^36,37^ Miglas Welay Gebregergis,^38^ Mesfin Gebrehiwot,^39^ Teferi Gebru Gebremeskel,^40,14^ Urge Gerema,^41^ Ebisa Zerihun Gindaba,^42^ Teklehaimanot Gereziher Haile,^43^ Demisu Zenbaba Heyi,^44^ Segun Emmanuel Ibitoye,^30^ Olayinka Stephen Ilesanmi,^45,46^ Mustapha Immurana,^47^ Chidozie Declan Iwu,^48^ Assefa N Iyasu,^43^ Rebuma Sorsa Jeben,^49^ Alelign Tasew Jema,^50^ Charity Ehimwenma Joshua,^51^ Gebisa Guyasa Kabito,^52^ Kehinde Kazeem Kanmodi,^53,54^ Molla Asnake Kebede,^55^ Manasi Kumar,^56,57^ Emmanuel Manu,^19^ Tesfahun Mekene Meto,^58^ Mathewos M Mekonnen,^59^ Hadush Negash Meles,^60^ Tadesse Misgana,^61^ Mustapha Mohammed,^62^ Prof Shafiu Mohammed,^63,64^ Ahmed Nuru Muhamed,^65^ Ogochukwu Janet Nzoputam,^66,67^ Onome Bright Oghenetega,^68^ Osaretin Christabel Okonji,^69^ Prof Andrew T Olagunju,^70,71^ Isaac Iyinoluwa Olufadewa,^72,73^ Gideon Olamilekan Oluwatunase,^74,75^ Prof Verner N Orish,^76,77^ Jennifer Rickard,^78,79^ Afeez Abolarinwa Salami,^16,53^ Prof Soraya Seedat,^80^ Ashenafi Kibret Sendekie,^81,82^ Mathilde Sengoelge,^1^ Migbar Mekonnen Sibhat,^83^ Yonatan Solomon,^84^ Prof Dan J Stein,^85^ Jacques Lukenze Tamuzi,^86,87^ Prof Elvis Enowbeyang Tarkang,^19,88^ Edosa Geta Tesfaye Gta,^89^ Temesgen Mohammed Toma,^90^ Aniefiok John Udoakang,^91^ Abate Dargie Wubetu,^92^ Prof Lucie Laflamme*,^1,93^ Mathilde  J Sengoelge*.^1,94^

*Joint senior authors

## **Affiliations**

^1^Department of Global Public Health, Karolinska Institute, Stockholm, Sweden; ^2^Department of Community Health, Eduardo Mondlane University, Maputo, Mozambique; ^3^Department of Clinical Governance and Quality Improvement, Aleta Wondo General Hospital, Aleta Wondo, Ethiopia; ^4^Department of Midwifery, Dilla University, Dilla, Ethiopia; ^5^Department of Biostatistics, Madda Walabu University, Bale Robe, Ethiopia; ^6^Department of Community Medicine, Babcock University, Ilishan-Remo, Nigeria; ^7^Department of Family and Community Health, University of Health and Allied Sciences, Ho, Ghana; ^8^Department of Population and Health, University of Cape Coast, Cape Coast, Ghana; ^9^Department of Health Promotion, Education and Behavior, University of South Carolina, Columbia, SC, USA; ^10^Department of Public Health, University of KwaZulu-Natal, Durban, South Africa; ^11^Department of HIV and Infectious Diseases, Jhpiego, Abuja, Nigeria; ^12^Department of Adolescent Research and Care, Adolescent Friendly Research Initiative and Care, Ado Ekiti, Nigeria; ^13^Department of Epidemiology, Jimma University, Jimma, Ethiopia; ^14^College of Medicine and Public Health, Flinders University, Adelaide, SA, Australia; ^15^Department of Oral and Maxillofacial Surgery, University of Ibadan, Ibadan, Nigeria; ^16^Department of Oral and Maxillofacial Surgery, University College Hospital, Ibadan, Ibadan, Nigeria; ^17^Lisbon Institute of Global Mental Health, Nova University of Lisbon, Lisbon, Nigeria; ^18^Clinical Pharmacy and Pharmacy Management, University of Nigeria Nsukka, Nsukka, Nigeria; ^19^Department of Population and Behavioural Sciences, University of Health and Allied Sciences, Ho, Ghana; ^20^Department of Electrical and Electronics Engineering Science, University of Johannesburg, Johannesburg, South Africa; ^21^Department of Public and Environmental Health, University of The Gambia, Banjul, The Gambia; ^22^Department of Epidemiology, University of Florida, Gainesville, FL, USA; ^23^Department of Animal Sciences, Obafemi Awolowo University, Ile-Ife, Nigeria; ^24^Department of Demography and Population Studies, University of Witwatersrand, Johannesburg, South Africa; ^25^School of Public Health, University of Zambia, Lusaka, Zambia; ^26^Department of Epidemiology and Global Health, Umeå University, Umea, Sweden; ^27^School of Public Health, Curtin University, Perth, WA, Australia; ^28^Department of Epidemiology, University of Gondar, Gondar, Ethiopia; ^29^USAID-JSI Digital Health Activity, Jimma University, Addis Ababa, Ethiopia; ^30^Department of Health Promotion and Education, University of Ibadan, Ibadan, Nigeria; ^31^Department of Epidemiology and Biostatistics, University of Health and Allied Sciences, Ho, Ghana; ^32^Faculty of Science and Health, University of Portsmouth, Hampshire, UK; ^33^Department of Microbiology, University of Medical Sciences, Ondo, Ondo, Nigeria; ^34^Department of Anesthesia, Cincinnati Children's Hospital Medical Center, Cincinnati, OH, USA; ^35^Department of Biotechnology, University of the Western Cape, Cape Town, South Africa; ^36^Department of Epidemiology and Medical Statistics, University of Ibadan, Ibadan, Nigeria; ^37^Research Centre for Healthcare and Community, Coventry University, Coventry, UK; ^38^Department of Midwifery, Adigrat University, Adigrat, Ethiopia; ^39^Department of Environmental Health, Wollo University, Dessie, Ethiopia; ^40^Department of Reproductive and Family Health, Axum College of Health Science, Axum, Ethiopia; ^41^Department of Public Health, Jimma University, Jimma, Ethiopia; ^42^Department of Nursing, Ethiopian Public Health Institute, Chiro, Ethiopia; ^43^Department of Nursing, Aksum University, Aksum, Ethiopia; ^44^Department of Public Health, Madda Walabu University, Robe, Ethiopia; ^45^West Africa RCC, Africa Centre for Disease Control and Prevention, Abuja, Nigeria; ^46^Department of Community Medicine, University College Hospital, Ibadan, Ibadan, Nigeria; ^47^Institute of Health Research, University of Health and Allied Sciences, Ho, Ghana; ^48^School of Health Systems and Public Health, University of Washington, Seattle, WA, USA; ^49^Department of Biomedical Sciences, Jimma University, Jimma, Ethiopia; ^50^Department of Public Health, Madda Walabu University, Goba, Ethiopia; ^51^Department of Economics, National Open University, Benin City, Nigeria; ^52^Department of Environmental and Occupational Health, University of Gondar, Gondar, Ethiopia; ^53^Faculty of Dentistry, University of Puthisastra, Phnom Penh, Cambodia; ^54^Office of the Executive Director, Cephas Health Research Initiative Inc, Ibadan, Nigeria; ^55^Department of Medicine, Mizan-Tepi University, Mizan Teferi, Ethiopia; ^56^Institute for Excellence in Health Equity, New York University, New York, NY, USA; ^57^Department of Psychiatry, University of Nairobi, Nairobi, Kenya; ^58^Department of Public Health, Arba Minch University, Arba Minch, Ethiopia; ^59^Department of Nursing, Salale University, Fiche, Ethiopia; ^60^Department of Medical Laboratory Sciences, Adigrat University, Adigrat, Ethiopia; ^61^Department of Psychiatry, Haramaya University, Harar, Ethiopia; ^62^QU Health, Qatar University, Doha, Qatar; ^63^Health Systems and Policy Research Unit, Ahmadu Bello University, Zaria, Nigeria; ^64^Heidelberg Institute of Global Health (HIGH), Heidelberg University, Heidelberg, Germany; ^65^Department of Nursing, Wolkite University, Wolkite, Ethiopia; ^66^Department of Physiology, University of Benin, Edo, Nigeria; ^67^Department of Physiology, Benson Idahosa University, Benin City, Nigeria; ^68^Department of Obstetrics and Gynecology, University of Ibadan, Ibadan, Nigeria; ^69^School of Pharmacy, University of the Western Cape, Cape Town, South Africa; ^70^Department of Psychiatry and Behavioural Neurosciences, McMaster University, Hamilton, ON, Canada; ^71^Department of Psychiatry, University of Lagos, Lagos, Nigeria; ^72^Slum and Rural Health Initiative Research Academy, Slum and Rural Health Initiative, Ibadan, Nigeria; ^73^Faculty of Public Health, University of Ibadan, Ibadan, Nigeria; ^74^Department of Anatomy, University of Medical Sciences, Ondo, Ondo, Nigeria; ^75^Department of Anatomy, Olabisi Onabanjo University, Sagamu, Nigeria; ^76^Department of Microbiology and Immunology, University of Health and Allied Sciences, Ho, Ghana; ^77^Sickle Cell Unit, Ho Teaching Hospital, Ho, Ghana; ^78^Department of Surgery, University of Minnesota, Minneapolis, MN, USA; ^79^Department of Surgery, University Teaching Hospital of Kigali, Kigali, Rwanda; ^80^Department of Psychiatry, Stellenbosch University, Cape Town, South Africa; ^81^Department of Clinical Pharmacy, University of Gondar, Gondar, Ethiopia; ^82^School of Pharmacy, Curtin University, Perth, WA, Australia; ^83^Department of Pediatrics and Child Health Nursing, Dilla University, Dilla, Ethiopia; ^84^Department of Nursing, Dire Dawa University, Dire Dawa, Ethiopia; ^85^SAMRC Unit on Risk and Resilience in Mental Disorders, University of Cape Town, Cape Town, South Africa; ^86^Department of Epidemiology, Stellenbosch University, Cape Town, South Africa; ^87^Department of Medicine, Northlands Medical Group, Omuthiya, Namibia; ^88^School of Nursing and Public Health, University of KwaZulu-Natal, Durban, South Africa; ^89^Department of Public Health, Wollega University, Nekemte, Ethiopia; ^90^Department of Public Health Emergency Management, South Ethiopia Region Public Health Institute, Jinka, Ethiopia; ^91^Department of Biosciences and Biotechnology, University of Medical Sciences, Ondo, Ondo, Nigeria; ^92^Department of Psychiatry, Debre Berhan University, Debre Berhan, Ethiopia; ^93^Institute for Social and Health Sciences, University of South Africa, Pretoria, South Africa; ^94^Department of Women and Child Health, Uppsala University, Uppsala, Sweden.

**Detailed information about individual author contributions**

### **Providing data or critical feedback on data sources**

Yohannes Habtegiorgis Abate, Richard Gyan Aboagye, Oluwafemi Atanda Adeagbo, Abiola Victor Victor Adepoju, Muktar Beshir Ahmed, Hubert Amu, Getnet Melaku Ayele, Amadou Barrow, Obasanjo Afolabi Bolarinwa, Malizgani Paul Chavula, Berecha Hundessa Demessa, Robert Kokou Dowou, Michael Ekholuenetale, Temitope Cyrus Ekundayo, Adewale Oluwaseun Fadaka, Adeniyi Francis Fagbamigbe, Teferi Gebru Gebremeskel, Teklehaimanot Gereziher Haile, Demisu Zenbaba Heyi, Segun Emmanuel Ibitoye, Olayinka Stephen Ilesanmi, Charity Ehimwenma Joshua, Manasi Kumar, Tesfahun Mekene Meto, Mathewos M Mekonnen, Mustapha Mohammed, Shafiu Mohammed, Sergio Keita Nhassengo, Ogochukwu Janet Nzoputam, Onome Bright Oghenetega, Andrew T Olagunju, Gideon Olamilekan Oluwatunase, Afeez Abolarinwa Salami, Migbar Mekonnen Sibhat, Yonatan Solomon, Elvis Enowbeyang Tarkang, Temesgen Mohammed Toma, and Abate Dargie Wubetu.

### **Developing methods or computational machinery**

Abiola Victor Victor Adepoju, Muktar Beshir Ahmed, Hubert Amu, Amadou Barrow, Michael Ekholuenetale, Adeniyi Francis Fagbamigbe, Teferi Gebru Gebremeskel, Teklehaimanot Gereziher Haile, Charity Ehimwenma Joshua, Mathewos M Mekonnen, Osaretin Christabel Okonji, and Andrew T Olagunju.

### **Providing critical feedback on methods or results**

Yohannes Habtegiorgis Abate, Mesfin Abebe, Richard Gyan Aboagye, Oluwafemi Atanda Adeagbo, Abiola Victor Victor Adepoju, Muktar Beshir Ahmed, Timothy Olukunle Aladelusi, Hubert Amu, Idowu Thomas Aruleba, Getnet Melaku Ayele, Amadou Barrow, Hameed Akande Bashiru, Obasanjo Afolabi Bolarinwa, Malizgani Paul Chavula, Berihun Assefa Dachew, Berecha Hundessa Demessa, Isaac Oluwafemi Dipeolu, Robert Kokou Dowou, Michael Ekholuenetale, Temitope Cyrus Ekundayo, Adewale Oluwaseun Fadaka, Adeniyi Francis Fagbamigbe, Miglas Welay Gebregergis, Mesfin Gebrehiwot, Urge Gerema, Ebisa Zerihun Gindaba, Teklehaimanot Gereziher Haile, Demisu Zenbaba Heyi, Segun Emmanuel Ibitoye, Olayinka Stephen Ilesanmi, Mustapha Immurana, Chidozie Declan Iwu, Assefa N Iyasu, Rebuma Sorsa Jeben, Alelign Tasew Jema, Charity Ehimwenma Joshua, Kehinde Kazeem Kanmodi, Molla Asnake Kebede, Manasi Kumar, Emmanuel Manu, Tesfahun Mekene Meto, Mathewos M Mekonnen, Hadush Negash Meles, Tadesse Misgana, Mustapha Mohammed, Shafiu Mohammed, Ahmed Nuru Muhamed, Sergio Keita Nhassengo, Ogochukwu Janet Nzoputam, Onome Bright Oghenetega, Osaretin Christabel Okonji, Andrew T Olagunju, Isaac Iyinoluwa Olufadewa, Gideon Olamilekan Oluwatunase, Jennifer Rickard, Afeez Abolarinwa Salami, Soraya Seedat, Ashenafi Kibret Sendekie, Mathilde Sengoelge, Migbar Mekonnen Sibhat, Yonatan Solomon, Jacques Lukenze Tamuzi, Elvis Enowbeyang Tarkang, Edosa Geta Tesfaye Gta, Temesgen Mohammed Toma, Aniefiok John Udoakang, and Abate Dargie Wubetu.

### **Drafting the work or revising it critically for important intellectual content**

Yohannes Habtegiorgis Abate, Mesfin Abebe, Kedir Hussein Abegaz, Olumide Abiodun, Kenneth Setorwu Adde, Oluwafemi Atanda Adeagbo, Muktar Beshir Ahmed, Timothy Olukunle Aladelusi, Deborah Oyine Aluh, Hubert Amu, Idowu Thomas Aruleba, Getnet Melaku Ayele, Amadou Barrow, Hameed Akande Bashiru, Malizgani Paul Chavula, Isaac Oluwafemi Dipeolu, Robert Kokou Dowou, Michael Ekholuenetale, Adewale Oluwaseun Fadaka, Adeniyi Francis Fagbamigbe, Miglas Welay Gebregergis, Urge Gerema, Teklehaimanot Gereziher Haile, Demisu Zenbaba Heyi, Segun Emmanuel Ibitoye, Olayinka Stephen Ilesanmi, Mustapha Immurana, Chidozie Declan Iwu, Assefa N Iyasu, Charity Ehimwenma Joshua, Gebisa Guyasa Kabito, Kehinde Kazeem Kanmodi, Manasi Kumar, Lucie Laflamme, Emmanuel Manu, Mathewos M Mekonnen, Hadush Negash Meles, Tadesse Misgana, Mustapha Mohammed, Shafiu Mohammed, Sergio Keita Nhassengo, Ogochukwu Janet Nzoputam, Onome Bright Oghenetega, Osaretin Christabel Okonji, Andrew T Olagunju, Gideon Olamilekan Oluwatunase, Verner N Orish, Jennifer Rickard, Afeez Abolarinwa Salami, Soraya Seedat, Mathilde Sengoelge, Migbar Mekonnen Sibhat, Yonatan Solomon, Dan J Stein, Jacques Lukenze Tamuzi, Temesgen Mohammed Toma, Aniefiok John Udoakang, and Abate Dargie Wubetu.

### **Managing the estimation or publications process**

Sergio Keita Nhassengo, Mathilde Sengoelge.
