# Supplementary material for: Trends and demographic differences in interpersonal violence against children in sub-Saharan Africa: findings from the 1990–2019 Global Burden of Disease Study
Source: BMJ Open. 2025 Apr 28;15(4):e083070. doi: 10.1136/bmjopen-2023-083070 (PMC12039018; doi:10.1136/bmjopen-2023-083070)
Supplement: Supplementary file 3 [file bmjopen-2023-083070.reviewer_comments.pdf]

PEER REVIEW HISTORY

BMJ Open publishes all reviews undertaken for accepted manuscripts. Reviewers are asked to complete a checklist review form and are provided with free text boxes to elaborate on their assessment. These free text comments are reproduced below.

ARTICLE DETAILS

Title (Provisional)

Trends and demographic differences in interpersonal violence to children in sub-Saharan Africa: findings from the 1990-2019 Global Burden of Disease Study

Authors

Nhassengo, Sergio; LAFLAMME, Lucie; Sengoelge, Mathilde; Child Interpersonal Collaborator Group, GBD

VERSION 1 - REVIEW

**Reviewer**	**1**
**Name**	**Cooper, Daniel**
**Affiliation**	**University of South Carolina, Psychology**
**Date**	**19-Jul-2024**
**COI**	**None**

Overall

- This study involved examining trends in child victimization in sub-Saharan Africa between 2009 and 2019. It compared rates by country, region, and type of violence exposure. It also examined whether trends varied based on country income level and alcohol consumption. Results could aid in improving scientific understanding of child interpersonal violence across sub-Saharan Africa. The study has numerous strengths, such as the use of a large dataset that allowed for country comparisons, and a clear contribution to a gap in the literature. However, there are several considerations that merit attention before publication. Copyediting is needed throughout in addition to revising colloquial language (e.g., "description of the like" on p. 14). I include more detailed feedback below.

Introduction

- It would be helpful to elaborate on the socio-ecological framework that guided this study. It is referenced throughout the study, but little explanation is given.
- More background on how alcohol consumption relates to victimization would be helpful to better justify your specific focus on this risk factor

Results

- Authors provide a table (i.e., supplemental table 5) breaking down the study findings by gender. Was this one of the goals of the study? There is no discussion of these findings anywhere in the manuscript. As rates of child victimization often differ by gender, it might be informative to discuss these results and their implications.

Discussion

- I am curious what explanations authors may provide for the considerable improvements in child victimization rates over time. What might be driving these trends? This is important to understand to keep trends moving in a positive direction.
- Authors state the importance of income inequality as a predictor of child victimization. How would this lens help to explain some of your study findings regarding income/SDI? The connection could be made more explicit.
- It might be helpful to provide the range of child victimization rates found in previous studies, even if they were attained using different methods and data sources.

Reviewer **2**

Name **Banerjee , Ankush**

Affiliation **All India Institute of Hygiene and Public Health, Preventive and Social Medicine**

Date **18-Aug-2024**

COI **None**

I want to congratulate the authors for such am extensive research work on a very important topic in the current scenario. The manuscript has been written lucidly and elaborately.

Just some minor issues

• How was the age standardisation of death rates was done can be elaborated in the methodology.

• Pearson correlation coefficient can be done when the data set was normally distributed. How was it checked. Otherwise Spearman coefficient has to be assessed

• Figure 4: the scatter plot can show a line of fit for easy understanding of the trend.

• I have some doubts regarding the estimation of uncertainty intervals. How was it estimated? Was it different from confidence intervals?

• Some of the references need to be written as per standard guidelines (Vancouver or as per journal guidelines)

**Reviewer**	**3**
**Name**	**Blangis , Flora**
**Affiliation**	**Inserm U1153**
**Date**	**12-Nov-2024**
**COI**	**No**

Many thanks for the invitation to review this manuscript. The authors used Global Burden of Disease data to examine trends in interpersonal violence against children in sub-Saharan Africa from 1990 to 2019. The paper is well structured and addresses an urgent public health issue by examining the incidence, mortality and disability-adjusted life years due to violence against children across different regions and demographic subgroups. The authors' work is crucial for advancing child protection policies in line with international goals.

The following are minor points for your consideration:

- In the abstract, the results related to alcohol consumption should be discussed in the conclusion.

- In the conclusion of the abstract, the phrase “prevention agenda that tailors region-wide drivers of violence and country specific factors” is not directly supported by any specific results.

- Although the authors refer to an external source for the definition of violence, including a brief definition in the methods section would clarify how 'violence' and its forms were defined for this research. The same applies to the DALYs.

- The authors used data spanning from 1990 to 2019. Since 2024, data are available up to 2021, which would be particularly interesting to include in order to examine trends during the COVID-19 pandemic period.

- Sexual violence in this study does not give details on female genital mutilation, although this could provide valuable insights, as it is a relevant form of gender-based violence with severe long-term consequences.

- Including age group analyses directly in the main results and discussion sections would strengthen the study. The manuscript includes age-specific data, but could benefit from further emphasizing these findings in the main discussion.

- A detailed breakdown of DALYs by specific causes of violence, if available, would provide valuable insights. The GBD database typically allows for this level of detail, which could show which specific types of violence or injury contribute most to the DALY burden across different age groups and regions.

- In the results, the authors use the term “association” when reporting correlations. It would be more precise to refer to these as “significant” or “non-significant correlations” to avoid

implying causality. Additionally, a statement in the discussion clarifying that these correlations do not imply causation would strengthen the interpretation.

- There are some very minor typos in the draft. e.g “children`s death” page 8 line 39 and “(sum of years of life lost (YLLs) and years lived with disability (YLDs)” a parenthesis is missing page 7 line 45.

VERSION 1 - AUTHOR RESPONSE

Editor comments	Authors' Responses	Edited in manuscript
**Reviewer 1**		
Introduction		
1. It would be helpful to elaborate on the socio-ecological framework that guided this study. It is referenced throughout the study, but little explanation is given.	Comment addressed.	P. 4
2. More background on how alcohol consumption relates to victimization would be helpful to better justify your specific focus on this risk factor.	Comment addressed.	P. 4-5
Results		
3. Authors provide a table (i.e., supplemental table 5) breaking down the study findings by gender. Was this one of the goals of the study? There is no discussion of these findings anywhere in the manuscript. As rates of child victimization often differ by gender, it might be informative to discuss these results and their implications.	Comment addressed. Child interpersonal violence deaths by sex results are now added in the results and the results are discussed.	Pag. 9-10
Discussion		
4. I am curious what explanations authors may provide for the considerable improvements in child victimization rates over time. What might be driving these trends? This is important to understand to keep trends moving in a positive direction.	We have addressed this comment in the discussion and referenced key documents.	P. 11
5. Authors state the importance of income inequality as a predictor of child victimization. How would this lens help to explain some of your study findings regarding income/SDI? The connection could be made more explicit.	Comment addressed.	P. 4-5
6. It might be helpful to provide the range of child victimization rates found in previous	Other refences of victimization rates in	P.5, 11 & 12

studies**,** even if they were attained using different methods and data sources.	previous studies are now mentioned.	
**Reviewer 2: Dr. Ankush Banerjee**		
1.How was the age standardisation of death rates was done can be elaborated in the methodology.	Information on this point is incorporated in the text.	P. 7
2. Pearson correlation coefficient can be done when the data set was normally distributed. How was it checked. Otherwise Spearman coefficient has to be assessed**.**	The data is not normally distributed, for that reason, as suggested by the reviewer, Spearman coefficient was assessed to analyse the correlation between the variables.	P. 7 & 10
3. Figure 4: the scatter plot can show a line of fit for easy understanding of the trend.	Line of fit added in figure 4.	P. 7-8 (Figures file)
4. Some of the references need to be written as per standard guidelines (Vancouver or as per journal guidelines)	All references have now been formatted per the journal guidelines.	
5. I have some doubts regarding the estimation of uncertainty intervals. How was it estimated? Was it different from confidence intervals?	The GBD study uses uncertainty intervals in their estimation. Generally, confidence intervals and uncertainty intervals are treated differently. Uncertainty intervals include all sources of uncertainty, including those arising from systematic biases and measurement error. 95% UI in these studies is wider than 95% CI as the UI covers an interval (Lower value-Upper value), with a certain probability.<br>In contrast, reported confidence intervals are based solely on the variation observed in sample data and CI covers a point. This information has now been added to the methods.	P. 7
**Reviewer 3: Dr. Flora Blangis**		
1.In the abstract, the results related to alcohol consumption should be discussed in the conclusion.	Elaborated further on in the discussion as per the suggestions of reviewer 1 and I hope this will be	P. 4, 12 & 13

	to the reviewer satisfaction.	
2. In the conclusion of the abstract, the phrase “prevention agenda that tailors region-wide drivers of violence and country specific factors” is not directly supported by any specific results.	Point taken. The phrase has been removed.	P. 1
3.Although the authors refer to an external source for the definition of violence, including a brief definition in the methods section would clarify how 'violence' and its forms were defined for this research. The same applies to the DALYs.	All relevant definitions are now included in the methods, aligned to the ones used in the GBD study definition.	P. 6
4. The authors used data spanning from 1990 to 2019. Since 2024, data are available up to 2021, which would be particularly interesting to include in order to examine trends during the COVID-19 pandemic period.	We agree this would be interesting to cover the consequences of the COVID-19 pandemic period, but that this warrants a separate article to address it properly.	P. 11
5. Sexual violence in this study does not give details on female genital mutilation, although this could provide valuable insights, as it is a relevant form of gender-based violence with severe long-term consequences.	This aspect is interested indeed. However, the GBD study does not provide rates specific to female genital mutilation at the global level. This is a very important topic in Sub Saharan Africa and the lack of these data is now stressed in the limitations section.	P. 3
6.Including age group analyses directly in the main results and discussion sections would strengthen the study. The manuscript includes age-specific data but could benefit from further emphasizing these findings in the main discussion.	Comment addressed.	P. 9 & 12
7.A detailed breakdown of DALYs by specific causes of violence, if available, would provide valuable insights. The GBD database typically allows for this level of detail, which could show which specific types of violence or injury contribute most to the DALY burden across different age groups and regions.	We fully agree. Yet, these data are not available at the regional level, which hinders any detailed breakdown. This has now been underlined in the limitations.	P. 3
8. In the results, the authors use the term “association” when reporting correlations. It would be more precise to refer to these as “significant” or “non-significant correlations” to avoid implying causality. Additionally, a statement in the discussion clarifying that	The term “association” was replaced by “correlations” and clarified that these correlations do not imply causation.	P. 10-12

these correlations do not imply causation would strengthen the interpretation.		
9. There are some very minor typos in the draft. e.g "children`s death" page 8 line 39 and "(sum of years of life lost (YLLs) and years lived with disability (YLDs)" a parenthesis is missing page 7 line 45.	Typos corrected.	P. 8

VERSION 2 - REVIEW

Reviewer **1**

Name **Cooper, Daniel**

Affiliation **University of South Carolina, Psychology**

Date **19-Mar-2025**

COI

I commend the authors for their hard work in revising the manuscript. It is much improved. I have no further comments at this time.

Reviewer **2**

Name **Banerjee , Ankush**

Affiliation **All India Institute of Hygiene and Public Health, Preventive and Social Medicine**

Date **14-Mar-2025**

COI

The authors have addressed the comments adequately.
